# Mediterranean-style diet in pregnant women with metabolic risk factors (ESTEEM): A pragmatic multicentre randomised trial

**DOI:** 10.1371/journal.pmed.1002857

**Published:** 2019-07-23

**Authors:** Bassel H. Al Wattar, Julie Dodds, Anna Placzek, Lee Beresford, Eleni Spyreli, Amanda Moore, Francisco J. Gonzalez Carreras, Frances Austin, Nilaani Murugesu, Tessa J. Roseboom, Maira Bes-Rastrollo, Graham A. Hitman, Richard Hooper, Khalid S. Khan, Shakila Thangaratinam, for the ESTEEM study group

**Affiliations:** 1 BARC (Barts Research Centre for Women’s Health), Women's Health Research Unit, Blizard Institute, Barts and the London School of Medicine and Dentistry, Queen Mary University of London, London, United Kingdom; 2 Warwick Medical School, University of Warwick, Coventry, United Kingdom; 3 Pragmatic Clinical Trials Unit, Blizard Institute, Barts and the London School of Medicine and Dentistry, Queen Mary University of London, London, United Kingdom; 4 Diabetes & Nutritional Sciences Division, Faculty of Life Sciences & Medicine, King's College, London, United Kingdom; 5 Maternity Dietetic Service, Women’s and Children’s Services, Barts NHS Trust, London, United Kingdom; 6 Department of Clinical Epidemiology, Biostatistics and Bioinformatics, Academic Medical Center, Amsterdam, the Netherlands; 7 Department of Obstetrics and Gynaecology, Academic Medical Center, Amsterdam, the Netherlands; 8 Department of Preventive Medicine and Public Health, University of Navarra, Pamplona, Spain; 9 CIBERobn, Instituto de Salud Carlos III, Madrid, Spain; 10 IDISNA, Navarra Institute for Health Research, Pamplona, Spain; 11 Barts Diabetes and Obesity Research Group, Blizard Institute, Barts and the London School of Medicine and Dentistry, Queen Mary University of London, London, United Kingdom; 12 Multidisciplinary Evidence Synthesis Hub (mEsh), Blizard Institute, Barts and the London School of Medicine, Queen Mary University London, London, United Kingdom; London School of Hygiene and Tropical Medicine, UNITED KINGDOM

## Abstract

**Background:**

Pregnant women with metabolic risk factors are at high risk of complications. We aimed to assess whether a Mediterranean-style diet reduces adverse pregnancy outcomes in high-risk women.

**Methods and findings:**

We conducted a multicentre randomised trial in 5 maternity units (4 in London and 1 in Birmingham) between 12 September 2014 and 29 February 2016. We randomised inner-city pregnant women with metabolic risk factors (obesity, chronic hypertension, or hypertriglyceridaemia) to a Mediterranean-style diet with high intake of nuts, extra virgin olive oil, fruits, vegetables, nonrefined grains, and legumes; moderate to high consumption of fish; low to moderate intake of poultry and dairy products; low intake of red and processed meat; and avoidance of sugary drinks, fast food, and food rich in animal fat versus usual care. Participants received individualised dietary advice at 18, 20, and 28 weeks’ gestation. The primary endpoints were composite maternal (gestational diabetes or preeclampsia) and composite offspring (stillbirth, small for gestational age, or admission to neonatal care unit) outcomes prioritised by a Delphi survey. We used an intention-to-treat (ITT) analysis with multivariable models and identified the stratification variables and prognostic factors a priori.

We screened 7,950 and randomised 1,252 women. Baseline data were available for 593 women in the intervention (93.3% follow-up, 553/593) and 612 in the control (95.6% follow-up, 585/612) groups. Over a quarter of randomised women were primigravida (330/1,205; 27%), 60% (729/1,205) were of Black or Asian ethnicity, and 69% (836/1,205) were obese. Women in the intervention arm consumed more nuts (70.1% versus 22.9%; adjusted odds ratio [aOR] 6.8, 95% confidence interval [CI] 4.3–10.6, *p* ≤ 0.001) and extra virgin olive oil (93.2% versus 49.0%; aOR 32.2, 95% CI 16.0–64.6, *p* ≤ 0.001) than controls; increased their intake of fish (*p* < 0.001), white meat (*p* < 0.001), and pulses (*p* = 0.05); and reduced their intake of red meat (*p* < 0.001), butter, margarine, and cream (*p* < 0.001). There was no significant reduction in the composite maternal (22.8% versus 28.6%; aOR 0.76, 95% CI 0.56–1.03, *p* = 0.08) or composite offspring (17.3% versus 20.9%; aOR 0.79, 95% CI 0.58–1.08, *p* = 0.14) outcomes. There was an apparent reduction in the odds of gestational diabetes by 35% (aOR 0.65, 95% CI 0.47–0.91, *p* = 0.01) but not in other individual components of the composite outcomes. Mothers gained less gestational weight (mean 6.8 versus 8.3 kg; adjusted difference −1.2 Kg, 95% CI −2.2 to −0.2, *p* = 0.03) with intervention versus control. There was no difference in any of the other maternal and offspring complications between both groups. When we pooled findings from the Effect of Simple, Targeted Diet in Pregnant Women With Metabolic Risk Factors on Pregnancy Outcomes (ESTEEM) trial with similar trials using random effects meta-analysis, we observed a significant reduction in gestational diabetes (odds ratio [OR] 0.67, 95% CI 0.53–0.84, I^2^ = 0%), with no heterogeneity (2 trials, 2,397 women). The study’s limitations include the use of participant reported tools for adherence to the intervention instead of objective biomarkers.

**Conclusions:**

A simple, individualised, Mediterranean-style diet in pregnancy did not reduce the overall risk of adverse maternal and offspring complications but has the potential to reduce gestational weight gain and the risk of gestational diabetes.

**Trial registration:**

ClinicalTrials.gov NCT02218931.

## Abstract 2

Bassel H. Al Wattar and colleagues reveal reduced levels of gestational diabetes for obese pregnant women who adopt a Mediterranean diet.

## Author summary

**Why was this study done?:**

- A Mediterranean-style diet reduced the risk of type 2 diabetes and cardiovascular complications in randomised trials involving the general population with risk factors.
- To date, no trial has evaluated the effects of a Mediterranean-style diet in pregnancy on maternal and offspring outcomes.

**What did the researchers do and find?:**

- In the Effect of Simple, Targeted Diet in Pregnant Women With Metabolic Risk Factors on Pregnancy Outcomes (ESTEEM) study, 1,252 pregnant women with metabolic risk factors from 5 inner-city United Kingdom hospitals were randomly allocated to follow a Mediterranean-style diet (supplemented with mixed nuts and extra virgin olive oil) or routine antenatal care, and the overall maternal (gestational diabetes or preeclampsia) and offspring outcomes (small-for-gestational age, stillbirth, or admission to the neonatal care unit) were assessed.
- Pregnant women allocated to the Mediterranean-style diet increased their intake of key components of this diet and gained on average 1.25 kg less weight than those who had routine care.
- There were no statistically significant reductions in overall maternal and offspring complications in pregnant women who followed a Mediterranean diet compared with usual care. Of the individual outcomes, the risk of gestational diabetes apparently decreased by 35% with the intervention.
- A meta-analysis of ESTEEM study results with a similar trial showed a 33% reduction in gestational diabetes but no effect on other outcomes.

**What do these findings mean?:**

- It is feasible to deliver a Mediterranean-style diet to inner-city pregnant women and increase their uptake of relevant dietary components.
- Mediterranean-style diet in pregnancy did not improve the overall maternal and offspring outcomes but has the potential to prevent gestational diabetes.
- Future studies should assess the effect of in utero exposure to Mediterranean-style diet—particularly to nuts and olive oil—on childhood obesity, allergy, and asthma.

## Introduction

One in four mothers enter pregnancy with preexisting obesity, chronic hypertension, or hyperlipidaemia [1,2]. In addition to complications in pregnancy, these mothers and their babies are at long-term risk of diabetes and cardiovascular complications [3]. A Mediterranean-style diet, rich in mono- and polyunsaturated fatty acids, reduces the incidence of cardiovascular diseases in the nonpregnant population [4–6]. In pregnancy, such a diet has the potential to improve maternal and offspring outcomes by preventing gestational diabetes, preeclampsia, and foetal growth restriction [7–9].

In the general population, individuals at high risk of cardiovascular diseases are advised to follow specific dietary patterns such as Dietary Approaches to Stop Hypertension (DASH) [10]. Despite the publication of numerous randomised trials on diet and lifestyle interventions in pregnancy [11], no clear dietary recommendations have emerged to improve pregnancy outcomes for women with metabolic risk factors. This can be attributed to the variations in the interventions and the reporting of outcomes as well as the lack of robust evidence on effectiveness of the diet [12]. To implement complex dietary interventions in practice, we require clear definitions of dietary components accompanied by simple guidance to improve adherence to the diet [13–15]. It is particularly challenging to evaluate such interventions in multi-ethnic pregnant populations comprising high-risk women from varied ethnic and socioeconomic backgrounds.

We undertook a multicentre pragmatic randomised trial (Effect of Simple, Targeted Diet in Pregnant Women With Metabolic Risk Factors on Pregnancy Outcomes [ESTEEM]) to evaluate the effects of a Mediterranean-style diet (supplemented with mixed nuts and extra virgin olive oil), with individualised dietary advice on maternal and offspring outcomes in pregnant women with metabolic risk factors, compared with routine antenatal care.

## Methods

### Trial oversight

The ESTEEM trial was approved by the NHS Research Ethics Committee (UK IRAS Reference No. 14/EE/1048). The Trial Steering Committee (TSC) provided independent supervision on all aspects of the trial conduct according to established principles and approved all protocol modifications (S1 Text) [16]. An independent Data Monitoring Committee (DMC) overviewed the trial’s data management and analysis. The California Walnut Commission and Blue Diamond Growers donated the walnuts, hazelnuts, and almonds without any influence on the study design, conduct, analysis, interpretation, or reporting. Extra virgin olive oil was purchased from industrial suppliers by the trial sponsor and was provided to the participants in the intervention group for free. The last author assumes responsibility for the completeness and accuracy of the data and analyses and for the fidelity of the trial to the protocol.

### Study design

A randomised controlled trial that recruited women from 5 inner-city maternity units in the UK (4 in London, 1 in Birmingham) between 12 September 2014 and 29 February 2016. The trial was prospectively registered with clinicaltrials.gov (NCT02218931), and the protocol was previously published by Al Wattar and colleagues [17].

### Participants

Pregnant women were recruited if they were at least 16 years of age, less than 18 weeks’ gestation with a singleton pregnancy, able to consume nuts and olive oil, and proficient in written and spoken English. We excluded participants with a history of preexisting diabetes, gestational diabetes, chronic renal disease, or autoimmune disease, or if they were taking lipid-altering drugs such as statins at the time of booking. After recruitment, we randomised women with metabolic risk factors such as obesity (body mass index [BMI] ≥ 30 kg/m^2^), raised serum triglycerides (≥1.7 mmol/L) or chronic hypertension (≥140 mm Hg systolic blood pressure [BP] or ≥90 mm Hg diastolic BP) to the intervention or control arms. We employed the cut-off values used to define metabolic risk in the general population because the tests were done in early pregnancy, reflective of the preconception risk status [18].

### Randomisation

Pregnant women received information about the trial alongside the invitation letter for their first antenatal booking appointment (S2 Text). After obtaining consent from eligible women, we collected baseline information on BP, BMI, and lipid profile (triglycerides, low-density, high-density, and very low-density cholesterol) at their booking visit. Women with at least one of the prespecified metabolic risk factors were randomised to the 2 arms of the trial in a 1:1 ratio via a password-protected online data management system. We used minimisation (with a random element to ensure allocation concealment) to balance the groups for maternal BMI, parity, and ethnicity.

### Intervention

The ESTEEM intervention was based on a Mediterranean-style diet. The key components of this diet included high intake of nuts, extra virgin olive oil, fruit, vegetables, nonrefined grains, and legumes; moderate to high consumption of fish; low to moderate intake of poultry and dairy products such as yoghurt and cheese; low consumption of red meat and processed meat; and avoidance of sugary drinks, fast food, and food rich in animal fat [19]. To promote their intake in pregnancy, we provided participants in the intervention arm with mixed nuts (30 g/day of walnuts, hazelnuts, and almonds) and extra virgin olive oil (0.5 L/week) as the main sources of cooking fat (S3 Text).

The trial dietitian and trained researchers delivered the intervention over 3 face-to-face sessions, which included a personalised one-on-one session at 18 weeks’ gestation, and 2 further group sessions at 20 and 28 weeks using prepiloted presentations (S3 Text) [17]. In the first visit, we used the 24-hour food recall technique to identify any changes that were needed in the participants’ diet to follow a Mediterranean-style pattern. We made the intervention culturally sensitive by providing cooking advice through a bespoke recipe book (S4 Text). We incorporated elements of the Mediterranean diet into the local cuisine by codesigning the recipes with community teams (food-academy.co.uk). Where possible, we involved women’s partners to participate in these sessions. In between the face-to-face sessions, we followed up with the women twice with phone calls at 24 and 32 weeks’ gestation to reinforce the dietary goals and to assess their general health. We used the number of sessions attended (at 18, 20, and 28 weeks’ gestation) as a marker of adherence.

We undertook an internal pilot phase to determine the dietary intake of pregnant women using a validated Food Frequency Questionnaire (FFQ) for Mediterranean diet and a modified short questionnaire (ESTEEM Q) that was previously validated for adherence to the Mediterranean diet in a nonpregnant population [20]. Details of the methods used to validate the FFQ against the 24-hour dietary recall and against the ESTEEM Q have been published elsewhere by Al Wattar and colleagues [17]. Subsequently, we used the ESTEEM Q to assess the intake of dietary components at 20, 24, 28, 32, and 36 weeks (S5 Text) [21].

Participants allocated to the control group received dietary advice as per UK national recommendations for antenatal care and weight management in pregnancy [22–24]. In addition to folic acid and vitamin D supplementation [24], mothers in both groups who were considered to be at high risk of preeclampsia received low dose aspirin (75 mg) [23]. Both groups completed questionnaires at 36 weeks’ gestation or at delivery to assess their level of physical activity (International Physical Activity Questionnaires [IPAQ]), gastrointestinal symptoms, and quality of life including the health thermometer (EQ-5D; S5 Text) [25].

### Outcome measures

The primary endpoints were a composite maternal outcome combining gestational diabetes or preeclampsia and a composite offspring outcome combining stillbirth, small-for-gestational-age fetus, or admission to the neonatal care unit [17]. The individual components of the composite were identified by a Delphi survey and were rated to be critically important in the care of pregnant women [26]. Gestational diabetes was defined as either a fasting venous glucose value of 5.1 mmol/L or more, or 2-hour values of 8.5 mmol/L or more following a 75-g oral glucose tolerance test (OGTT) as per the modified International Association of Diabetes and Pregnancy Study Group (IADPSG) criteria [27]. All participants were screened for gestational diabetes with OGTT as per national guidelines [28]. Preeclampsia included the following: new onset preeclampsia defined as onset of hypertension (systolic BP ≥ 140 mm Hg or diastolic BP ≥ 90 mmHg, in at least 2 readings, taken 4–6 hours apart) and new onset proteinuria (spot urine protein creatinine ratio [PCR] > 30 mg/mmol or 24-hour urine protein > 300 mg or 2+ or more on urine dipstick) after 20 weeks’ gestation; and superimposed preeclampsia in women with chronic hypertension or chronic proteinuria (S6 Text) [23]. Small-for-gestational age fetus included babies with birth weight less than 10th percentile on the customised charts that were adjusted for gestation at delivery, maternal height, weight, parity, and ethnicity [29].

The initial primary endpoint was preeclampsia. Following the planned internal pilot, the independent TSC recommended the primary endpoint be extended to include composite maternal outcome and composite offspring outcome. The committee considered both gestational diabetes and preeclampsia to be major burdens facing women with metabolic risk factors, and that a large trial on Mediterranean diet in pregnancy should evaluate these maternal and the relevant offspring outcomes ranked as critically important to clinical care in a Delphi survey of researchers [26]. This decision was made independent of the trial investigators, before completion of data collection and development of the statistical analysis plan. The change of outcomes was approved by the Research Ethics Committee and was incorporated in the published protocol. The funders and sponsor of the study had no involvement in the revision of endpoint.

Our secondary maternal outcomes included gestational diabetes, preeclampsia, gestational weight gain, maternal admission to high dependency or intensive care unit, antepartum haemorrhage, mode of delivery, preterm delivery, and maternal anaemia. Our secondary offspring outcomes included stillbirths, neonatal deaths, small-for-gestational age fetus (<10th percentile), admission to the neonatal care unit, birth weight, and hypoxic ischaemic encephalopathy. The maternal intake of food groups, levels of triglycerides, levels of high-density lipoproteins (HDLs), the ratio of triglycerides to HDL, and the levels of non–high-density lipoprotein (non-HDL) cholesterol were compared between the 2 groups. A dedicated research staff recorded outcomes from clinical notes and from hospital electronic records following delivery.

### Statistical analysis

We estimated the composite maternal and composite offspring outcomes to be observed in 24% of women with metabolic risk factors [30] and expected the intervention to reduce their occurrence by 30% [4]. We calculated that we needed to analyse data from 982 women to ensure an 80% power to detect this change at the 5% significance level. To allow for a loss of 20% at follow-up, we planned to randomise 1,230 women.

The primary analysis was conducted on an intention-to-treat (ITT) basis. Baseline demographics and clinical characteristics were summarised as percentages for categorical variables, mean (standard deviation) for normally distributed continuous variables, and median (interquartile range) for non-normally distributed variables. Participants who were enrolled in error were excluded post randomisation. Women who experienced miscarriages and terminations of pregnancy after randomisation were not included in any analysis of the offspring outcomes. We reported the effect of the intervention on composite maternal and foetal outcomes as adjusted odds ratio (aOR) with 95% confidence interval (CI) using a multivariable logistic regression model. In addition to the minimisation factors, we adjusted the primary analysis for other factors determined a priori (maternal age, history of gestational diabetes in a previous pregnancy, stillbirth in a previous pregnancy, family history of hypertensive disorder or diabetes, and the centre of recruitment) [21,28,22].

We repeated the ITT analysis for the primary composite outcomes in subgroups specified a priori for women with and without obesity, chronic hypertension, and raised triglycerides and tested for any interactions. The individual components of the primary outcome, as well as other secondary outcomes, were analysed using multivariable logistic regression for binary outcomes and linear regression for continuous outcomes, with a normalizing transformation when necessary. When a continuous outcome was also assessed at baseline, we adjusted for this as an additional covariate. We only included participants with nonmissing outcome in the primary analysis. This approach is unbiased if the data were ‘missing at random’, i.e., missingness for the outcome is related to the observed covariates. We investigated the sensitivity of our conclusions to this assumption, when more than 5% of data for the primary outcome were missing, by imputing missing outcomes under alternative assumptions and by re-estimating the treatment effect.

For validation of the FFQ against the 24-hour dietary recall and the ESTEEM Q, we specified a priori that the agreement using intraclass correlation coefficient (ICC) was good for nutrient values if the score was above 0.5, acceptable if between 0.49 and 0.2 and poor if below 0.2 [31]. For Kappa statistics, we considered the agreement to be good for κ > 0.6, acceptable for κ 0.6–0.2, and poor for κ < 0.2 [31]. Quartile cross-classification for agreement was considered to be good if ≥50% were in the same quartile and ≤10% were in the opposite quartile. The agreement was judged as poor if <50% were in the same quartile and >10% were in the opposite quartile. Values in between these ranges were judged to denote moderate agreement [31].

The study statisticians and the research team were blinded to the analyses and interpretation of results. An independent statistician provided unblinded summaries and reports using computer code to the DMC. All analyses for the trial were conducted using STATA version 12 or higher (StataCorp, College Station, TX, 2012).

We inferred the findings of the ESTEEM trial in the context of available evidence by systematically searching the literature (MEDLINE and EMBASE from inception till January 2019) for similar trials on Mediterranean diet in pregnancy. We extracted the data and pooled the results using random effects meta-analysis and reported the effects as an odds ratio (OR) with 95% CI and heterogeneity using I^2^ estimates [32].

### Patient and public involvement

We sought input from pregnant women at the trial design stage by conducting a survey of their views on the feasibility and acceptability of the planned intervention. The TSC had a lay representative from Action on Preeclampsia Charity (APEC; S1 Text).

## Results

**Fig 1:**
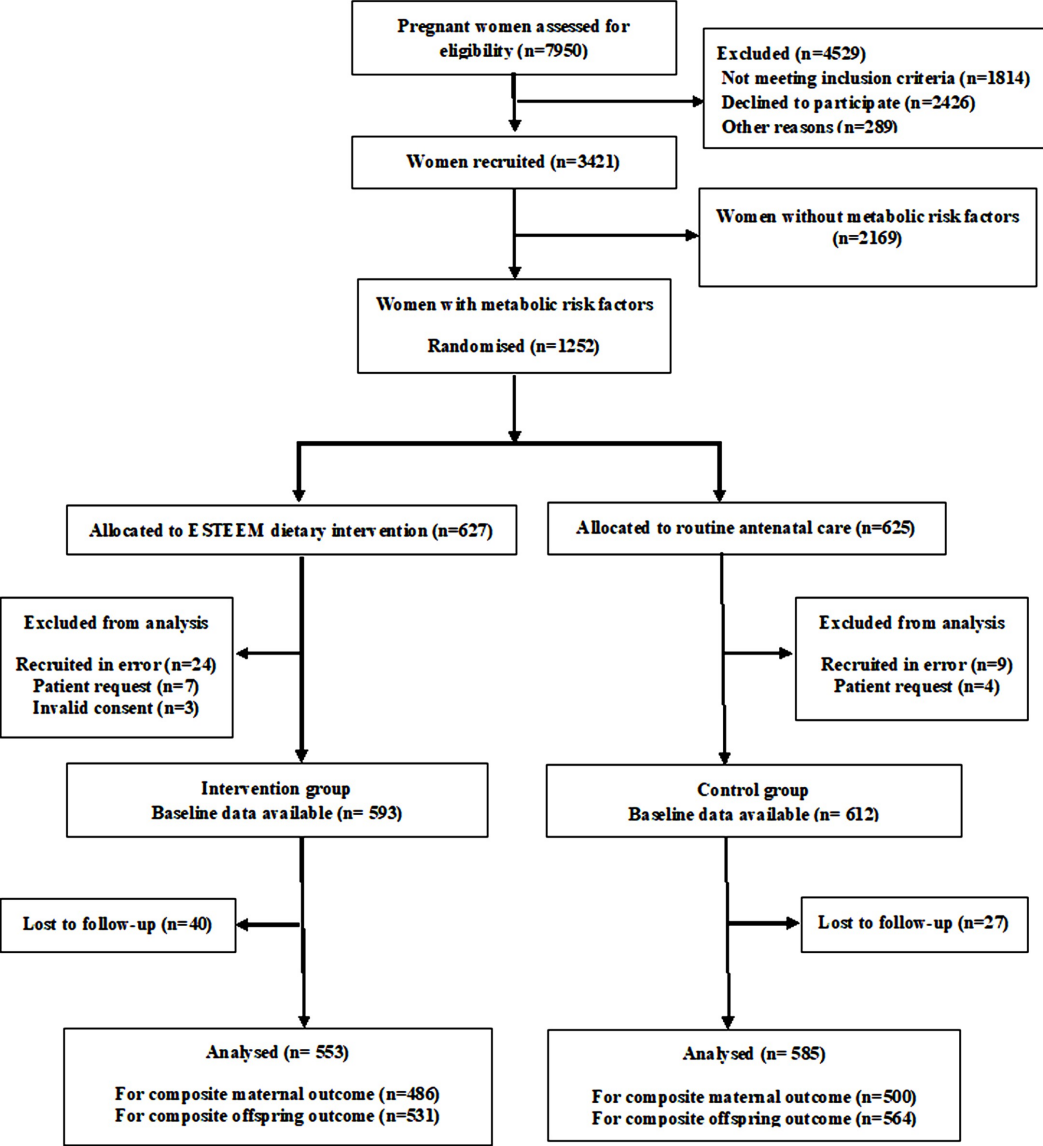
Enrolment, randomisation, and follow-up of participants in the ESTEEM trial. ESTEEM, Effect of Simple, Targeted Diet in Pregnant Women With Metabolic Risk Factors on Pregnancy Outcomes.

We screened 7,950 women, recruited 3,421, and randomised 1,252 women with metabolic risk factors to the intervention (*n* = 627) or to the control group (*n* = 625). Baseline data were available for 99% (1,237/1,252) of the randomised participants; 93% (553/593) in the intervention and 96% (585/612) in the control groups were followed up and included in the analysis. Fig 1 depicts the enrolment, randomisation, and follow-up of participants in the ESTEEM trial.

A third of the randomised women were primigravida (330/1,205; 27%), 60% were Black or Asian (729/1,205), 69% (836/1,205) were obese, 47% (515/1,090) had raised triglycerides at booking, and 5% (58/1,129) entered pregnancy with chronic hypertension (Table 1).

**Table 1:** Baseline characteristics of participants in the ESTEEM trial on Mediterranean-style diet in pregnancy.

Maternal characteristics (*N* intervention; control)	Intervention group Mean (SD); *n* (%)	Control group Mean (SD); *n* (%)
**Maternal age (584; 610)**		
Age years (mean SD) Age > 40 years	31.4 (5.2); 23 (3.9%)	30.9 (5.2); 19 (3.1%)
**Gravidity (593; 612)**		
Primigravida Multigravida	162 (27.3%) 431 (72.7%)	168 (27.5%) 444 (72.5%)
**Ethnicity (593; 612)**		
White Asian Black Other	217 (36.6%) 257 (43.3%) 97 (16.4%) 22 (3.7%)	217 (35.5%) 270 (44.1%) 105 (17.2%) 20 (3.3%)
BMI (kg/m^2^) (593; 612)		
Normal (18.5–24.9) Overweight (25.0–29.9) Obese (30.0–39.9)	84 (14.2%) 99 (16.7%) 410 (69.1%)	84 (13.7%) 102 (16.7%) 426 (69.6%)
**Medical history**		
Chronic hypertension (554; 575) Previous history of preeclampsia (575; 600) Previous history of gestational diabetes (563; 585) Previous stillbirth or neonatal death (571; 598) Previous admission to ITU/HDU (530; 557) Family history of hypertension/preeclampsia (537; 536) Family history of diabetes (541; 555)	27 (4.9%) 21 (3.7%) 15 (2.7%) 8 (1.4%) 5 (0.9%) 274 (51.0%) 276 (51.0%)	31 (5.4%) 29 (4.8%) 22 (3.8%) 14 (2.3%) 10 (1.8%) 276 (51.5%) 303 (54.6%)
**Serum lipids**		
Triglycerides mmol/L (532; 558) (mean SD) HDL mmol/L (531; 557) (mean SD) Ratio of triglycerides to cholesterol (527; 553) Non-HDL mmol/L (529; 554) (mean SD)	1.6 (0.7) 1.7 (0.5) 0.3 (0.2) 3.2 (0.8)	1.7 (0.8) 1.6 (0.4) 0.3 (0.2) 3.3 (1.6)
**Diet**		
ESTEEM Q score (337; 210) (mean SD)	5.0 (1.9)	5.0 (1.9)
**Physical activity**		
MET (minutes per week)/week (406; 241) (mean SD)	2,579.5 (3,335.9)	2,591.7 (3,306.9)
**Health**		
Health thermometer (400; 222) (mean SD)	67.4 (18.7)	71.8 (18.7)

**Abbreviations:** ESTEEM Q, Effect of Simple, Targeted Diet in Pregnant Women With Metabolic Risk Factors on Pregnancy Outcomes questionnaire; HDL, high-density lipoprotein; HDU, high dependency unit; ITU, intensive treatment unit; LDL, low-density lipoprotein; MET, metabolic equivalent of task; non-HDL, non–high-density lipoprotein; SD, standard deviation

Three-quarters (410/553, 74%) of women in the intervention group attended at least one of the planned intervention sessions. Women were able to receive their nuts and olive oil even when they missed a session. Mothers allocated to the intervention versus control group consumed significantly more nuts (70.1% versus 22.9%, aOR 6.8, 95% CI 4.3–10.6, *p* ≤ 0.001) and extra virgin olive oil (93.2% versus 49.0%, aOR 32.2, 95% CI 16.0–64.6, *p* ≤ 0.001); increased their intake of key components of the Mediterranean diet such as fish (*p* < 0.001), white meat (*p* < 0.001), and pulses (*p* = 0.05); and consumed less red meat (*p* < 0.001) and less butter, margarine, and cream (*p* < 0∙001). There were no differences between the groups in their intake of other food groups or their physical activity (metabolic equivalent of task [MET]) (mean difference [MD] −0.2, 95% CI −0.1 to 0.51, *p* = 0.19; Table 2).

**Table 2:** Changes in the reported dietary intake, physical activity, and health gastrointestinal symptoms in pregnant women in the ESTEEM trial.

Maternal diet, symptoms, and physical activity *N* (intervention; control)	Intervention	Control	Crude OR or MD (95% CI)	Crude *p*-value	aOR^#^ or adjusted MD (95% CI)	Adjusted *p*-value
Dietary intake						
Olive oil as main fat source* (280; 298)	261 (93.2%)	146 (49.0%)	14.30 (8.52–24.01)	<0.001	32.19 (16.03–64.62)	<0.001
≥4 tbsp. of olive oil/day* (273; 296)	63 (23.1%)	28 (9.5%)	2.87 (1.78–4.64)	<0.001	2.81 (1.55–5.09)	<0.001
≥3 nut servings/week* (274; 292)	192 (70.1%)	67 (22.9%)	7.86 (5.40–11.45)	<0.001	6.75 (4.28–10.62)	<0.001
≥3 fish or shellfish servings/week* (276; 297)	101 (36.6%)	67 (22.6%)	1.98 (1.37–2.86)	<0.001	2.57 (1.57–4.21)	<0.001
Preferential consumption of chicken or turkey instead of veal, pork, hamburger, or sausage* (254; 279)	221 (87.0%)	224 (80.3%)	1.64 (1.03–2.63)	0.038	2.34 (1.26–4.35)	0.007
≤1 red meat, processed meat, or red meat products servings/day* (241; 278)	206 (85.5%)	156 (56.1%)	4.60 (3.00–7.07)	<0.001	3.42 (1.99–5.86)	<0.001
≤1 butter, margarine, or cream servings/day* (268; 291)	164 (61.2%)	115 (39.5%)	2.41 (1.72–3.39)	<0.001	2.41 (1.55–3.75)	<0.001
≤1 sugary drinks/day* (269; 295)	149 (55.4%)	121 (41.0%)	1.79 (1.28–2.49)	<0.001	1.40 (0.92–2.15)	0.12
≥3 pulse servings/week* (275; 296)	103 (37.5%)	86 (29.1%)	1.46 (1.03–2.08)	0.033	1.56 (1.00–2.44)	0.048
≥3 fruit units/day* (276; 296)	142 (51.4%)	156 (52.7%)	0.95 (0.68–1.32)	0.76	1.10 (0.72–1.68)	0.66
≤3 times consumption of commercial sweets or pastries/week* (276; 292)	165 (59.8%)	151 (51.7%)	1.39 (1.00–1.94)	0.053	1.42 (0.92–2.19)	0.11
≥2 vegetable servings/day* (274; 293)	185 (67.5%)	189 (64.5%)	1.14 (0.81–1.62)	0.45	1.34 (0.85–2.11)	0.21
ESTEEM Q score (mean SD) (218; 255)	7.2 (2.0)	5.1 (2.0)	2.1 (1.7–2.4)	<0.001	2.0 (1.7–2.3)	<0.001
Maternal gastrointestinal symptoms						
Fullness of stomach (266; 251)	151 (56.8%)	157 (62.5%)	0.79 (0.55–1.12)	0.18	0.93 (0.60–1.43)	0.73
Bloatedness (267; 250)	76 (28.5%)	92 (36.8%)	0.68 (0.47–0.99)	0.04	0.61 (0.39–0.96)	0.03
Vomiting (266; 250)	35 (13.2%)	44 (17.6%)	0.71 (0.44–1.15)	0.16	0.61 (0.33–1.14)	0.12
Nausea (267; 252)	70 (26.2%)	83 (32.9%)	0.72 (0.50–1.06)	0.09	0.82 (0.52–1.31)	0.41
Indigestion (267; 250)	126 (47.2%)	110 (44.0%)	1.14 (0.80–1.61)	0.47	1.08 (0.69–1.69)	0.73
Constipation (267; 251)	70 (26.2%)	82 (32.7%)	0.73 (0.50–1.07)	0.11	0.73 (0.46–1.15)	0.17
Diarrhoea (265; 250)	32 (12.1%)	39 (15.6%)	0.74 (0.45–1.23)	0.25	0.60 (0.34–1.09)	0.09
Health and physical activity						
Physical activity (MET [minutes per week]) (mean SD) (262; 270)	6.9 (1.6)	6.7 (2.0)	0.24 (−0.07–0.56)	0.13	0.21 (−0.10–0.51)	0.19
Health thermometer (mean SD) (257; 252)	73.1 (16.9)	69.9 (18.6)	3.3 (0.2–6.4)	0.038	3.0 (0.1–5.9)	0.046

^#^OR: adjusted for the minimisation factors, age, history of previous gestational diabetes, family history of hypertensive disorders (hypertension and/or preeclampsia), family history of diabetes, history of stillbirth, and the recruitment centre.

*Score of 1 for each positive response

**Abbreviations:** CI, confidence interval; ESTEEM Q, Effect of Simple, Targeted Diet in Pregnant Women With Metabolic Risk Factors on Pregnancy Outcomes questionnaire; MD, mean difference; MET, metabolic equivalent of task;OR, odds ratio; SD, standard deviation; tbsp, tablespoon

### Outcomes

We did not observe a significant reduction in the odds of the composite maternal (aOR 0.76, 95% CI 0.56–1.03, *p* = 0.08) and composite offspring (aOR 0.79, 95% CI 0.58–1.08, *p* = 0.14) outcomes with Mediterranean-style diet compared with usual care. For the individual components of the maternal composite outcome, there was a significant reduction in the odds of gestational diabetes by 35% (aOR 0.65, 95% CI 0.47–0.91, *p* = 0.01) but no significant effect on preeclampsia (aOR 1.4, 95% CI 0.84–2.4, *p* = 0.19) in the intervention group. We did not observe a significant reduction in any of the individual components of the composite offspring outcome (small-for-gestational age fetus: aOR 0.78, 95% CI 0.53–1.15, *p* = 0.21; stillbirth: aOR 0.49, 95% CI 0.04–5.57, *p* = 0.56; and admission to the neonatal unit: aOR 0.79, 95% CI 0.53–1.18, *p* = 0.25; Table 3).

**Table 3:** Effects of Mediterranean-style diet in pregnancy on the primary composite maternal and composite offspring outcomes and their individual components.

Outcomes (*N* intervention; control)	Intervention *N* (%)	Control*N* (%)	Crude OR (95% CI)	Crude *p*-value	aOR* (95% CI)	Adjusted *p*-value
**Primary outcomes**						
Composite maternal outcome (486; 500)**	111 (22.8%)	143 (28.6%)	0.74 (0.55–0.98)	0.04	0.76 (0.56–1.03)	0.08
Composite offspring outcome (531; 564)^#^	92 (17.3%)	118 (20.9%)	0.79 (0.59–1.07)	0.13	0.79 (0.58–1.08)	0.14
**Individual components of the composite outcomes**						
Gestational diabetes (477; 497)	84 (17.6%)	124 (24.9%)	0.64 (0.47–0.88)	0.01	0.65 (0.47–0.91)	0.01
Preeclampsia (552; 585)	34 (6.2%)	27 (4.6%)	1.36 (0.81–2.28)	0.25	1.43 (0.84–2.43)	0.19
Small-for-gestational age fetus (531; 564)^$^^,^^#^	52 (9.8%)	69 (12.2%)	0.78 (0.53–1.14)	0.20	0.78 (0.53–1.15)	0.21
Stillbirth (533; 566)^#^	1 (0.2%)	2 (0.4%)	0.53 (0.05–5.86)	0.60	0.49 (0.04–5.57)	0.56
Admission to neonatal unit (533; 565)^#^	49 (9.2%)	64 (11.3%)	0.79 (0.54–1.17)	0.25	0.79 (0.53–1.18)	0.25

*aOR: adjusted for the minimisation factors, age, history of previous gestational diabetes, family history of hypertensive disorders (hypertension and/or preeclampsia), family history of diabetes, history of stillbirth and the recruitment centre.

** Women with gestational diabetes or with preeclampsia were considered to have the composite maternal outcome even if the other component of outcome was missing; for the composite outcome to be considered absent both components needed to be confirmed absent–otherwise the composite outcome was considered to be missing. Similar approach was used for the composite offspring outcome.

^$^Small for gestation age: <10th centile using customised charts adjusting for maternal height, weight, parity, gestation at delivery and ethnic origin

^#^Denominator for components of composite offspring outcome excludes women with miscarriage and termination of pregnancy.

**Abbreviations:** aOR, adjusted odds ratio; CI, confidence interval; MD, mean difference

Women allocated to the intervention gained less weight in pregnancy (MD −1.2 kg, 95% CI −2.2 to −0.2, *p* = 0.03) than the control group. There were no differences between the groups for other secondary outcomes (Table 4).

**Table 4:** Effects of Mediterranean-style diet in pregnancy on the secondary maternal and offspring outcomes in pregnant women with metabolic risk factors.

Outcomes *N* (intervention; control)	Intervention *N* (%)	Control *N* (%)	Crude OR or MD (95% CI)	Crude *p*-value	aOR^#^ or adjusted MD (95% CI)	Adjusted *p*-value
**Maternal clinical outcomes**						
Gestational weight gain (kg) (mean, SD) (230; 238)	6.8 (5.6)	8.3 (6.4)	−1.5 (−2.6 to −0.4)	0.01	−1.2 (−2.2 to −0.2)	0.03
Preterm delivery <37 weeks (545; 579)	52 (9.5%)	64 (11.1%)	0.85 (0.58 to 1.3)	0.41	0.82 (0.55 to 1.22)	0.33
Preterm delivery <34 weeks (545; 579)	23 (4.2%)	26 (4.5%)	0.94 (0.53 to 1.66)	0.82	0.92 (0.51 to 1.67)	0.79
Antepartum haemorrhage (548; 580)	9 (1.6%)	13 (2.2%)	0.73 (0.31 to 1.72)	0.47	0.70 (0.29 to 1.72)	0.44
Caesarean section (539; 571)	175 (32.6%)	176 (30.8%)	1.08 (0.84 to 1.39)	0.56	1.06 (0.8 to 1.37)	0.65
Anaemia (547; 578)	114 (20.8%)	129 (22.3%)	0.92 (0.69 to 1.22)	0.55	0.91 (0.66 to 1.23)	0.53
Admission to HDU or ITU (527; 566)	18 (3.4%)	24 (4.2%)	0.80 (0.43 to 1.49)	0.48	0.79 (0.42 to 1.50)	0.48
**Offspring outcomes**						
Hypoxic ischaemic encephalopathy (531; 561)	2 (0.4%)	4 (0.7%)	0.53 (0.10 to 2.89)	0.46	0.56 (0.09 to 3.46)	0.53
Neonatal death (532; 566)	3 (0.6%)	1 (0.2%)	3.20 (0.33 to 30.90)	0.31	3.93 (0.33 to 46.10)	0.28
Birth weight (g) (mean, SD) (531; 565)	3,340.1 (623.1)	3,277.6 (599.4)	62.4 (−10.0 to 134.9)	0.09	56.0 (−15.4 to 127.4)	0.12
Very small for gestational age (customised) (531; 564)*	17 (3.2%)	21 (3.7%)	0.86 (0.45 to 1.64)	0.64	0.84 (0.43 to 1.63)	0.60
Large for gestational age (customised) (531; 564)*	73 (13.7%)	64 (11.3%)	1.25 (0.87 to 1.78)	0.23	1.23 (0.86 to 1.78)	0.26
Small for gestational age (population based) (531; 564)**	61 (11.5%)	86 (15.2%)	0.72 (0.51 to 1.03)	0.07	0.73 (0.51 to 1.04)	0.08
Very small for gestational age (population based) (531; 564)**	30 (5.6%)	33 (5.9%)	0.96 (0.58 to 1.60)	0.89	0.96 (0.57 to 1.61)	0.87
Large for gestational age (population based) (531; 564)**	59 (11.1%)	61 (10.8%)	1.03 (0.71 to 1.51)	0.88	1.01 (0.69 to 1.49)	0.94
**Maternal laboratory outcomes (mean, SD)**						
Triglycerides (mmol/L) (217; 257)	3.0 (1.3)	2.9 (1.3)	0.08 (−0.15 to 0.31)	0.50	0.04 (−0.15 to 0.22)	0.70
HDL (mmol/L) (221; 258)	1.8 (0.5)	1.8 (0.5)	0.03 (−0.06 to 0.12)	0.54	0.02 (−0.05 to 0.09)	0.51
Ratio of triglycerides to cholesterol (215; 255)	0.5 (0.2)	0.5 (0.2)	0.01 (−0.03 to 0.04)	0.67	0.01 (−0.02 to 0.04)	0.72
Non-HDL (mmol/L) (219; 256)	4.4 (1.3)	4.3 (1.3)	0.03 (−0.21 to 0.27)	0.82	0.01 (−0.18 to 0.19)	0.93

^#^aOR: adjusted for the minimisation factors, age, history of previous gestational diabetes, family history of hypertensive disorders (hypertension and/or preeclampsia), family history of diabetes, history of stillbirth and the recruitment centre

*Customised birth weight centile adjusted for maternal height, weight, parity, gestational age at delivery and ethnic origin

** Population based birth weight centile

**Abbreviations:** aOR, adjusted odds ratio; CI, confidence interval; HDL, high-density lipoprotein;HDU, high dependency unit; ITU, intensive treatment unit; MD, mean difference; non-HDL, non–high-density lipoprotein; SD, standard deviation

A sensitivity analysis that imputed missing outcomes under varying departures from the missing-at-random assumption found the results to be qualitatively unchanged even when the OR for missing versus nonmissing outcomes was as high as 3 or as low as 0.33 (S1 Fig).

There were no major differences in the treatment effect for composite maternal or offspring outcomes within the subgroups of maternal obesity, chronic hypertension, or raised triglycerides (Table 5).

**Table 5:** Subgroup analysis on the effects of Mediterranean-style diet in pregnancy on the composite maternal and offspring outcomes and their individual components.

Outcomes *N* (intervention; control)	Intervention n (%)	Control n (%)	aOR* (95% CI)	Adjusted *p*-value
**Composite maternal outcome**				
Obese (348; 352)	81 (23.3%)	104 (29.5%)	0.72 (0.50–1.02)	0.55
Not obese (138; 148)	30 (21.7%)	39 (26.4%)	0.88 (0.50–1.57)	
Raised triglycerides (199; 212)	50 (25.1%)	71 (33.5%)	0.68 (0.43–1.08)	0.59
Normal triglycerides (243; 245)	51 (21.0%)	61 (24.9%)	0.81 (0.52–1.26)	
Chronic hypertension (30; 27)	10 (33.3%)	14 (51.9%)	0.60 (0.19–1.89)	0.67
No chronic hypertension (448; 461)	100 (22.3%)	125 (27.1%)	0.78 (0.57–1.07)	
**Composite offspring outcome**				
Obese (373; 392)	61 (16.4%)	86 (21.9%)	0.69 (0.48–1.01)	0.20
Not obese (158; 172)	31 (19.6%)	32 (18.6%)	1.08 (0.61–1.89)	
Raised triglycerides (223; 242)	38 (17.0%)	45 (18.6%)	0.94 (0.58–1.52)	0.37
Normal triglycerides (258; 270)	46 (17.8%)	63 (23.3%)	0.69 (0.45–1.07)	
Chronic hypertension (35; 28)	16 (45.7%)	6 (21.4%)	3.08 (0.97–9.77)	0.02
No chronic hypertension (487; 523)	76 (15.6%)	108 (20.7%)	0.72 (0.52–1.00)	
**Preeclampsia**				
Obese (386; 409)	26 (6.7%)	18 (4.4%)	1.65 (0.88–3.11)	0.40
Not obese (166; 176)	8 (4.8%)	9 (5.1%)	0.99 (0.37–2.69)	
Raised triglycerides (230; 252)	11 (4.8%)	11 (4.4%)	1.13 (0.47–2.71)	0.91
Normal triglycerides (270; 280)	18 (6.7%)	16 (5.7%)	1.21 (0.59–2.46)	
Chronic hypertension (36; 30)	7 (19.4%)	2 (6.7%)	3.62 (0.65–20.01)	0.25
No chronic hypertension (507; 542)	27 (5.3%)	24 (4.4%)	1.26 (0.71–2.24)	
**Gestational diabetes**				
Obese (341; 349)	61 (17.9%)	92 (26.4%)	0.58 (0.40–0.86)	0.27
Not obese (136; 148)	23 (16.9%)	32 (21.6%)	0.88 (0.47–1.65)	
Raised triglycerides (195; 212)	41 (21.0%)	63 (29.7%)	0.64 (0.39–1.04)	0.86
Normal triglycerides (238; 242)	36 (15.1%)	50 (20.7%)	0.68 (0.41–1.11)	
Chronic hypertension (27; 27)	5 (18.5%)	13 (48.1%)	0.28 (0.07–1.07)	0.19
No chronic hypertension (442; 458)	78 (17.6%)	107 (23.4%)	0.70 (0.50–1.00)	
**Small for gestational age**^**$**^^**#**^				
Obese (375; 397)	33 (8.8%)	51 (12.8%)	0.65 (0.41–1.03)	0.28
Not obese (160; 174)	19 (11.9%)	21 (12.1%)	1.02 (0.52–2.00)	
Raised triglycerides (218; 239)	20 (9.2%)	23 (9.6%)	1.00 (0.53–1.91)	0.22
Normal triglycerides (256; 271)	27 (10.5%)	42 (15.5%)	0.60 (0.35–1.02)	
Chronic hypertension (35; 29)	10 (28.6%)	5 (17.2%)	2.02 (0.58–7.02)	0.09
No chronic hypertension (491; 529)	42 (8.6%)	66 (12.5%)	0.66 (0.43–0.99)	
**Admission to neonatal unit**^**#**^				
Obese (386; 409)	36 (9.3%)	45 (11.0%)	0.86 (0.53–1.38)	0.60
Not obese (166; 175)	13 (7.8%)	19 (10.9%)	0.67 (0.31–1.45)	
Raised triglycerides (230; 251)	20 (8.7%)	29 (11.6%)	0.74 (0.40–1.37)	0.73
Normal triglycerides (270; 280)	26 (9.6%)	32 (11.4%)	0.86 (0.49–1.52)	
Chronic hypertension (36; 30)	6 (16.7%)	2 (6.7%)	2.43 (0.43–13.79)	0.22
No chronic hypertension (507; 541)	43 (8.5%)	59 (10.9%)	0.78 (0.51–1.20)	

*aOR: adjusted for the minimisation factors, age, history of previous gestational diabetes, family history of hypertensive disorders (hypertension and/or preeclampsia), family history of diabetes, history of stillbirth and the recruitment centre.

^$^Small for gestation age: <10th percentile using customised charts adjusting for maternal height, weight, parity, gestation at delivery and ethnic origin

^#^Denominator for components of composite offspring outcome excludes women with miscarriage and termination of pregnancy

**Abbreviations:** aOR, adjusted odds ratio; CI, confidence interval

Participants in the intervention group reported better overall quality of life with higher health thermometer scores (MD 3.0, 95% CI 0.1–5.9, *p* = 0.046) than those in the control group (Table 2). Intake of a Mediterranean-style diet apparently reduced bloatedness (aOR 0.61, 95% CI 0.39–0.96, *p* = 0.03) in pregnancy but did not affect other symptoms such as nausea, vomiting, or indigestion (Table 2). One woman in the intervention arm had an allergic reaction to walnuts that resolved spontaneously. There were no serious adverse events in any of the participants.

Assessment of dietary intake of the participants using the FFQ showed good to acceptable agreement with the 24-hour dietary recall method for meat (ICC 0.56) and fish (ICC 0.52) and acceptable agreement for bread (ICC 0.46), vegetables (ICC 0.20), legumes (ICC 0.25), eggs (ICC 0.23), and pastries, cakes, and sweets (ICC 0.21). There was good quartile cross-classification agreement for the majority of food groups, except for eggs, legumes, olive oil, and nuts. ESTEEM Q demonstrated moderate to good agreement with the FFQ for the use of olive oil (κ 0.52), fruits (κ 0.36), butter or margarine (κ 0.33), sugary drinks (κ 0.50), fish (κ 0.30), commercial sweets (κ 0.35), and nuts (κ 0.36).

### Meta-analysis of randomised trials on Mediterranean diet in pregnancy

**Fig 2:**
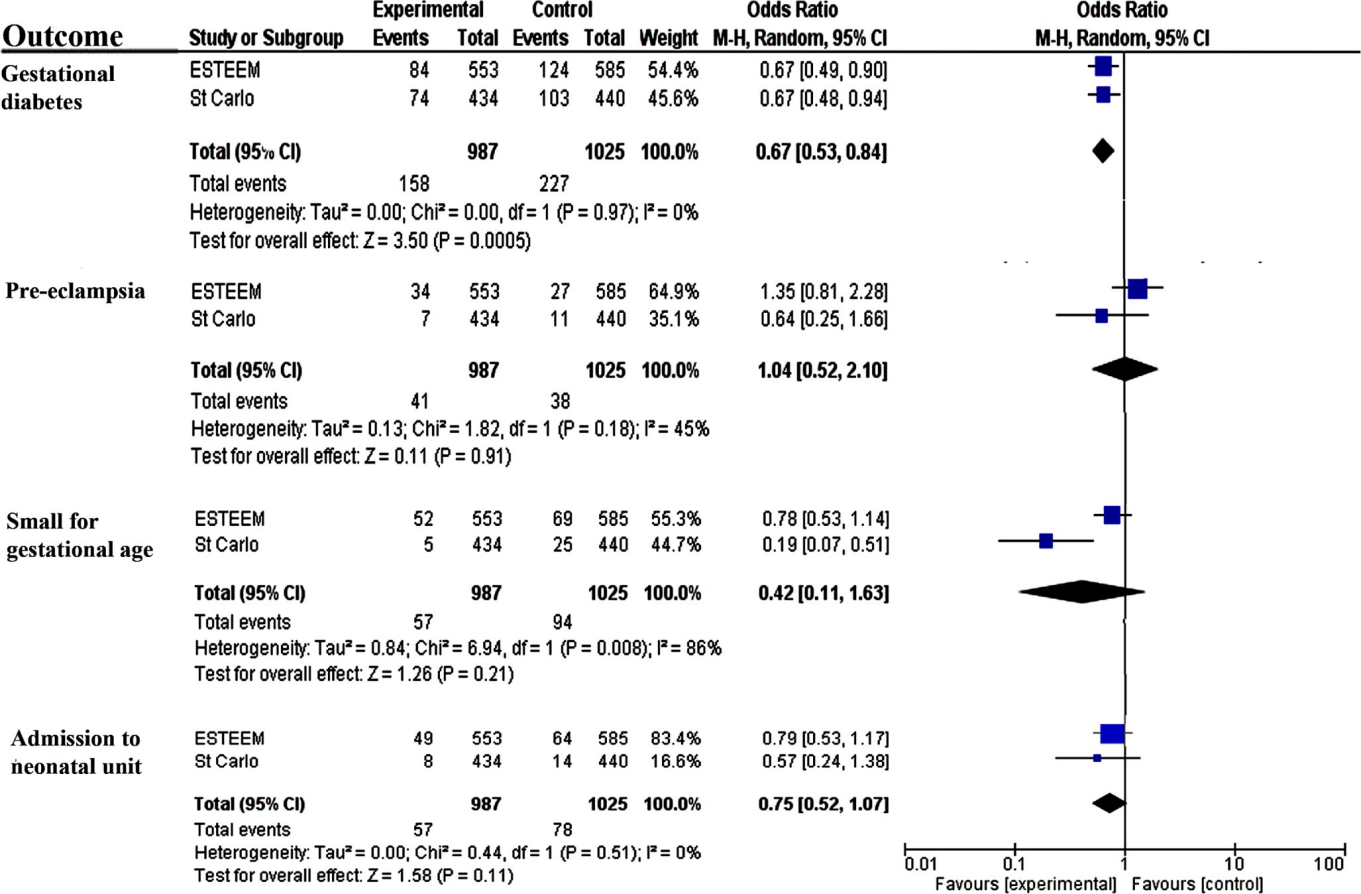
Meta-analysis of randomised trials on Mediterranean-style diet in pregnancy. CI, confidence interval; ESTEEM, Effect of Simple, Targeted Diet in Pregnant Women With Metabolic Risk Factors on Pregnancy Outcomes; M-H, Mantel-Haenszel.

Our literature search identified one randomised trial involving 874 unselected pregnant women from Spain (St. Carlos study) [33]. The women were mainly of Caucasian origin, and three-quarters (659/874, 75%) had normal BMI. The intervention was a Mediterranean diet supplemented with nuts and extra virgin olive oil. The pooled effect estimate (2 trials, 2,012 women) showed a consistent reduction in gestational diabetes (OR 0.67, 95% CI 0.53–0.84; I^2^ = 0%). The intervention did not reduce the rates of other individual components of the primary composite outcomes such as preeclampsia, small-for-gestational age fetus, or admission to the neonatal care unit. Fig 2 illustrates the forest plots for all conducted meta-analyses.

## Discussion

### Main findings

In the ESTEEM trial, we successfully implemented a Mediterranean-style diet in a multi-ethnic pregnant population of high-risk women. Mothers who followed the intervention significantly increased their consumption of nuts, olive oil, fish, white meat, and pulses and lowered their intake of red meat and butter or margarine compared with the usual care group. Overall, the intervention did not significantly reduce the composite maternal and offspring outcomes; still, it had an apparent protective effect to reduce the incidence of gestational diabetes and gestational weight gain.

### Strengths and weaknesses of the study

We registered and published the trial protocol prospectively [17]. Two-thirds of participants were from ethnic minority groups, who—in addition to being at risk of complications—are also usually difficult to engage in lifestyle interventions [34]. We used a simple, culturally sensitive, and easy to follow intervention; involved the partners and the wider family; and provided extra virgin olive oil and nuts. Three-quarters of participants (74%, 410/553) attended at least one of the planned intervention sessions, a rate that is comparable to other trials on diet-based interventions in pregnancy [30,35]. Mothers had the flexibility to receive olive oil and nuts even when they did not attend the face-to-face sessions. We evaluated the validity of the dietary assessment tools used within our study population for the key food groups in the Mediterranean diet. In women randomised to the intervention, we observed an increased intake of not only nuts and olive oil but also changes in the consumption of other key components of Mediterranean-style diet such as increased intake of fish, preferential intake of chicken and turkey over veal and sausages, and reduced consumption of red meat and butter compared with the control group, indicating adherence to the overall diet. Our choice of the individual components of the primary composite outcomes was based on a Delphi survey of experts who specified the outcomes to be critically important in the evaluation of lifestyle interventions in pregnancy [26]. The decision to add components to the original primary outcome was made by a committee independent to the trial team, without access to any data that may provide insight into the treatment effect.

We did not find a significant reduction in maternal composite outcome. The majority of our participants were multigravida (875/1,205; 73%). This may explain the relatively low incidence of preeclampsia (61/1,138; 5.3%), a condition more commonly observed in the first pregnancy. Our participants were from diverse ethnic and cultural backgrounds, and we were not able to take into account the variations in baseline dietary pattern. However, we ensured that the diet in the intervention arm was culturally appropriate and adapted to their individualised needs. We did not blind participants or researchers, which is difficult to achieve in complex dietary interventions [36] and cannot rule out a Hawthorne effect with women in the control group accessing the intervention [37]. Despite the overall high follow-up rate, we could not ascertain the outcome of gestational diabetes in 15% of participants. But this was below our expected attrition rate of 20%. We did not use additional measures such as biomarkers to objectively assess the intake of olive oil and nuts. We only obtained information on dietary intake in about 40% of participants in both groups, a proportion that is consistent with what was observed in similar dietary trials involving pregnant women [38]. Although variations in the clinical management of gestational diabetes and preeclampsia might have influenced the offspring outcomes, we consider this less likely because both groups were managed according to the UK national guidelines [23,28]. In our preplanned, primary analysis, we adopted a relatively simplistic approach to dealing with missing components of composite outcomes, which could have potentially introduced bias. We considered post hoc a more exclusive definition of each composite outcome that required all the respective components to be nonmissing, but the results were similar (for maternal composite outcome: aOR 0.71, 95% CI 0.52–0.97; composite offspring outcome: aOR 0.80, 95% CI 0.59–1.09). Our sensitivity analysis also suggested that our findings were robust to departures from a ‘missing at random’ assumption.

The primary end point changed after the start of the randomised trial. We consider the potential risk of bias to be minimal for the following reasons. The decision to change the endpoint by the independent TSC was based on the emerging evidence base on the burden of gestational diabetes and preeclampsia in women with metabolic risk factors, particularly in those from ethnic minority backgrounds with previous history of gestational diabetes. The TSC took into account observational evidence on Mediterranean diet in pregnancy and potential reductions in gestational diabetes [39,40], and the decision was made prior to completion of data collection and statistical analysis plan development and without knowledge of outcome data by allocated groups or any effect estimates. The changes in the endpoints were clearly documented in the revised protocol, in this paper, and were approved by the ethics committee Finally, we reported the effects of the intervention on both the composite outcomes and its individual components, which includes the original endpoint. We advise caution on the interpretation of the results in view of the revised endpoints, and this needs to be confirmed in future large trials.

### Comparison with other studies

In our individual participant data (IPD) meta-analysis (36 randomised trials, 12,526 women) on diet and physical activity in pregnancy, interventions reduced gestational weight gain and caesarean section rates but not other maternal or offspring outcomes [11]. For key outcomes such as gestational diabetes and preeclampsia, both aggregate and IPD meta-analyses were limited by the variations in the definitions between studies. Individual studies had either shown no benefit or had insufficient power to detect meaningful differences [11,30].

Unlike the effects of a Mediterranean diet in the general population [41], we did not observe any differences in the lipid levels between both arms in our study. We found a moderate reduction in the mean gestational weight gain with intervention compared with control, consistent with reports on the protective role of a Mediterranean diet against obesity and weight gain [42]. This could be attributed to the satiety achieved with a plant-based diet, increased fibre intake, and the low glycaemic index of promoted food groups such as the pulses [42]. But lowered weight gain did not translate into reductions in composite maternal and composite offspring outcomes. In the St. Carlos trial, the beneficial effect of a Mediterranean diet was observed for gestational diabetes but not for gestational weight gain. It is possible that this beneficial effect on gestational diabetes was mediated through the high intake of dietary polyphenols found in key components of the Mediterranean diet such as extra virgin olive oil and nuts, by reducing insulin resistance, stimulating insulin secretion, activating insulin receptors, modulating glucose release, and increasing the uptake of glucose in the insulin-sensitive tissues [43]. Although a Mediterranean diet has been shown to reduce high BP in the general population at risk [44], and high adherence to this diet reduced foetal growth restriction in observational studies [7], we did not observe any reductions in the risk of preeclampsia or any offspring outcome. This could be attributed to the fact that because placental remodelling occurs in early pregnancy, the dietary intervention might not have been started early enough or long enough to observe any benefit [45]. Furthermore, we observed fewer women with preeclampsia than expected in this high-risk group. Despite the increase in the sample size in the meta-analysis, there was no reduction in the risk of preeclampsia.

### Relevance to clinical practice and research

The results of ESTEEM combined with previous evidence show that supplementation of 30 g of mixed nuts per day and extra virgin olive oil can lower gestational weight gain and has strong potential to minimise risk of gestational diabetes. Delivering such dietary intervention is feasible as part of routine antenatal care, reflecting the pragmatic approach adopted in our trial. A definitive large-scale trial on a Mediterranean-style diet will need to assess both reduction in gestational diabetes and whether it translates to the prevention of type 2 diabetes in the mother in later life. The cost-effectiveness of following a Mediterranean-style diet also needs to be formally studied with a full economic evaluation. Although we did not find any differences between the groups in short-term offspring outcomes, we do not know the potential impact of in utero exposure to the various dietary components of the intervention on long-term outcomes such as childhood obesity and other conditions such as asthma and allergy disorders in the offspring. Furthermore, the long-term effects of the reduction in gestational diabetes in the mother on child outcomes also needs further evaluation. Future studies should assess the effect of in utero exposure to a Mediterranean-style diet on children, particularly to nuts and olive oil, on childhood obesity, allergy, and asthma [46].

### Conclusion

A simple, individualised, Mediterranean-style diet in pregnancy did not reduce the overall risk of adverse maternal and offspring complications but has the potential to reduce gestational weight gain and the risk of gestational diabetes.

## Supporting information

S1 Checklist CONSORT checklist. (DOC)

S1 Fig Analysis of effect of departures from the missing-at-random assumption on the primary end points. (DOCX)

S1 Table Analysis of health status and quality of life using the EQ-5D assessment tool for participants in the ESTEEM trial. ESTEEM, Effect of Simple, Targeted Diet in Pregnant Women With Metabolic Risk Factors on Pregnancy Outcomes.
(DOCX)

S1 Text Members of the ESTEEM study TSC and DMC. DMC, Data Monitoring Committee; ESTEEM, Effect of Simple, Targeted Diet in Pregnant Women With Metabolic Risk Factors on Pregnancy Outcomes; TSC, Trial Steering Committee.
(DOCX)

S2 Text Patient information sheet for the ESTEEM study. ESTEEM, Effect of Simple, Targeted Diet in Pregnant Women With Metabolic Risk Factors on Pregnancy Outcomes.
(DOCX)

S3 Text Intervention facts sheets and educational presentation for the ESTEEM trial. ESTEEM, Effect of Simple, Targeted Diet in Pregnant Women With Metabolic Risk Factors on Pregnancy Outcomes.
(DOCX)

S4 Text ESTEEM bespoke recipe book. ESTEEM, Effect of Simple, Targeted Diet in Pregnant Women With Metabolic Risk Factors on Pregnancy Outcomes.
(DOCX)

S5 Text ESTEEM Q, EQ5D, IPAQ, FFQ, and 24-hour recall questionnaires. ESTEEM Q, Effect of Simple, Targeted Diet in Pregnant Women With Metabolic Risk Factors on Pregnancy Outcomes questionnaire; FFQ, Food Frequency Questionnaire; IPAQ, International Physical Activity Questionnaires.
(DOCX)

S6 Text Definition of the primary outcomes. (DOCX)

## Abbreviations

aOR: adjusted odds ratio
APEC: Action on Preeclampsia Charity
BMI: body mass index
BP: blood pressure
CI: confidence interval
DASH: Dietary Approaches to Stop Hypertension
DMC: Data Monitoring Committee
ESTEEM: Effect of Simple, Targeted Diet in Pregnant Women With Metabolic Risk Factors on Pregnancy Outcomes
ESTEEM Q: ESTEEM questionnaire
FFQ: Food Frequency Questionnaire
HDL: high-density lipoprotein
HDU: high dependency unit
IADPSG: International Association of Diabetes and Pregnancy Study Group
ICC: intraclass correlation coefficient
IPAQ: International Physical Activity Questionnaires
IPD: individual participant data
ITT: intention-to-treat
ITU: intensive treatment unit
MD: mean difference
MET: metabolic equivalent of task
non-HDL: non–high-density lipoprotein
OGTT: oral glucose tolerance test
OR: odds ratio
PCR: protein creatinine ratio
TSC: Trial Steering Committee
