# Supplementary material for: Mediterranean-style diet in pregnant women with metabolic risk factors (ESTEEM): A pragmatic multicentre randomised trial
Source: PLoS Med. 2019 Jul 23;16(7):e1002857. doi: 10.1371/journal.pmed.1002857 (PMC6650045; doi:10.1371/journal.pmed.1002857)
Supplement: S1 Fig — (DOCX) [file pmed.1002857.s002.docx]

**S1 Fig:** Analysis of effect of departures from the missing at random assumption on the primary end points

A-Composite maternal outcome B- composite offspring outcome

**A-**
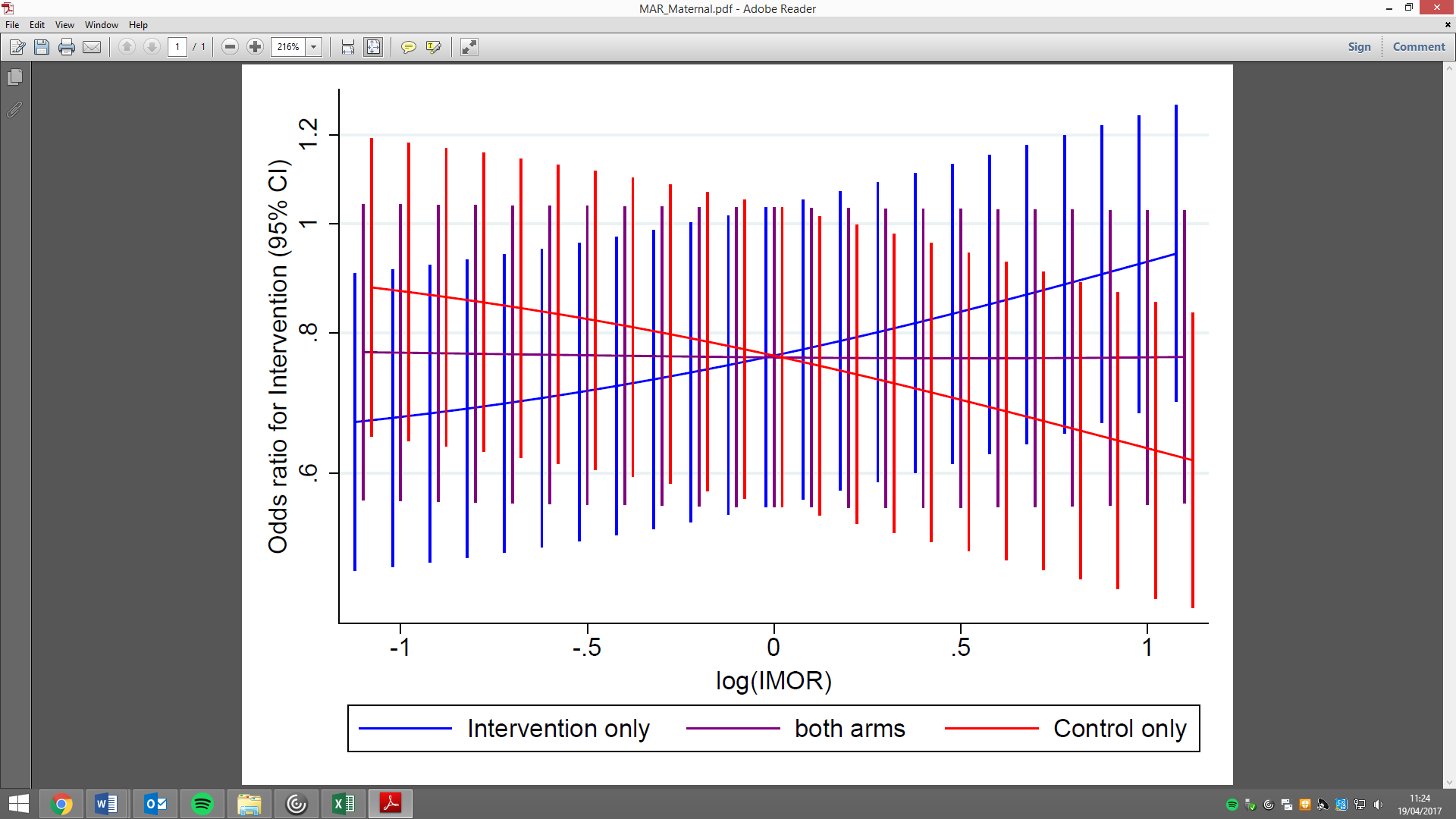
**B-**
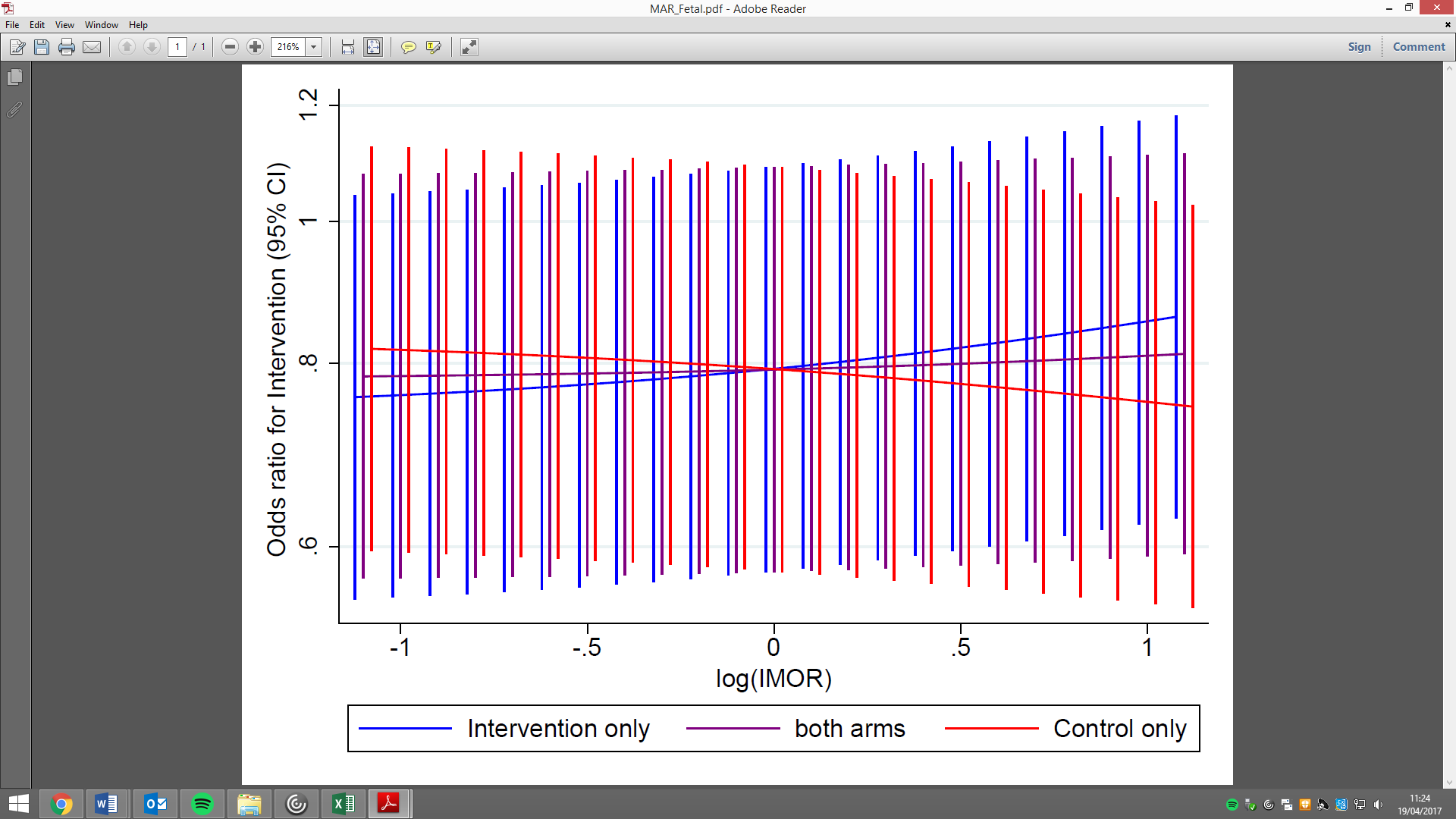


We detected >5% missingness in the primary endpoints (Composite Maternal outcome 18.2% and composite offspring outcome 6.2%). Our sensitivity analysis exploring departures from the Missing at Random (MAR) assumption was varied with Informative Missingness Odds Ratio (IMOR) between approximately 1/3 and 3, where a value of 1 equates to MAR (i.e. missing values are just as likely to be a success as observed values, adjusting for baseline covariates). The analysis was firstly undertaken in both arms, then separately within the intervention and control arms.

We concluded that our inferences about treatment effect on either primary end points were not sensitive to departures from the MAR assumption. We therefore analysed participants with complete outcomes only.
