# Supplementary material for: Mediterranean-style diet in pregnant women with metabolic risk factors (ESTEEM): A pragmatic multicentre randomised trial
Source: PLoS Med. 2019 Jul 23;16(7):e1002857. doi: 10.1371/journal.pmed.1002857 (PMC6650045; doi:10.1371/journal.pmed.1002857)
Supplement: S1 Table — ESTEEM, Effect of Simple, Targeted Diet in Pregnant Women With Metabolic Risk Factors on Pregnancy Outcomes. (DOCX) [file pmed.1002857.s003.docx]

**S1 Table:** Analysis of health status and quality of life using the EQ-5D assessment tool for participants in the ESTEEM trial

| Health State domain (n,%) | Intervention (n=553) | Control (n=585) | Crude Odds Ratio (95% CI) | Crude P Value | Adjusted Odds Ratio (95% CI) | Adjusted P Value |
| --- | --- | --- | --- | --- | --- | --- |
| Mobility | 83 (29.6%) | 84 (28.1%) | 1.08 (0.75, 1.55) | 0.68 | 1.06 (0.68, 1.64) | 0.80 |
| Self-Care | 25 (9.0%) | 36 (12.0%) | 0.72 (0.42, 1.23) | 0.23 | 0.73 (0.37, 1.46) | 0.37 |
| Usual Activities | 88 (31.7%) | 102 (34.0%) | 0.90 (0.63, 1.27) | 0.55 | 0.88 (0.57, 1.35) | 0.55 |
| Pain/Discomfort | 174 (62.4%) | 189 (63.2%) | 0.96 (0.69, 1.35) | 0.83 | 0.91 (0.60, 1.38) | 0.65 |
| Anxiety/Depression | 53 (19.1%) | 59 (19.7%) | 0.96 (0.63, 1.45) | 0.84 | 1.07 (0.65, 1.78) | 0.78 |
