# Supplementary material for: Mediterranean-style diet in pregnant women with metabolic risk factors (ESTEEM): A pragmatic multicentre randomised trial
Source: PLoS Med. 2019 Jul 23;16(7):e1002857. doi: 10.1371/journal.pmed.1002857 (PMC6650045; doi:10.1371/journal.pmed.1002857)
Supplement: S1 Text — DMC, Data Monitoring Committee; ESTEEM, Effect of Simple, Targeted Diet in Pregnant Women With Metabolic Risk Factors on Pregnancy Outcomes; TSC, Trial Steering Committee. (DOCX) [file pmed.1002857.s004.docx]

**S1 Text:** Members of the ESTEEM study trial steering committee and data monitoring committee.

1. Independent members of the trial steering committee:
2. Dr Harold Gee (Chair), Consultant Obstetrician (retired)
3. Ms Anne Marie Barnard, CEO of Action Pre-eclampsia (APEC)
4. Dr Louise Goff, Lecturer in Nutritional Sciences, King’s College London
5. Dr Andrew Ewer, Reader in Neonatal paediatrics, University of Birmingham
6. Independent members of the data monitoring committee:
7. Professor Gordon Smith, Professor and Head of Department, Obstetrics and Gynaecology, University of Cambridge
8. Lee Middleton, Senior Medical Statistician, Birmingham Clinical Trials Unit
9. Professor Gerben ter Riet, Associate Professor, Academic Medical Centre, Amsterdam
