# Supplementary figures and images for: Mediterranean-style diet in pregnant women with metabolic risk factors (ESTEEM): A pragmatic multicentre randomised trial
Source: PLoS Med. 2019 Jul 23;16(7):e1002857. doi: 10.1371/journal.pmed.1002857 (PMC6650045; doi:10.1371/journal.pmed.1002857)

**S2 Text:** Patient information sheet for the ESTEEM study


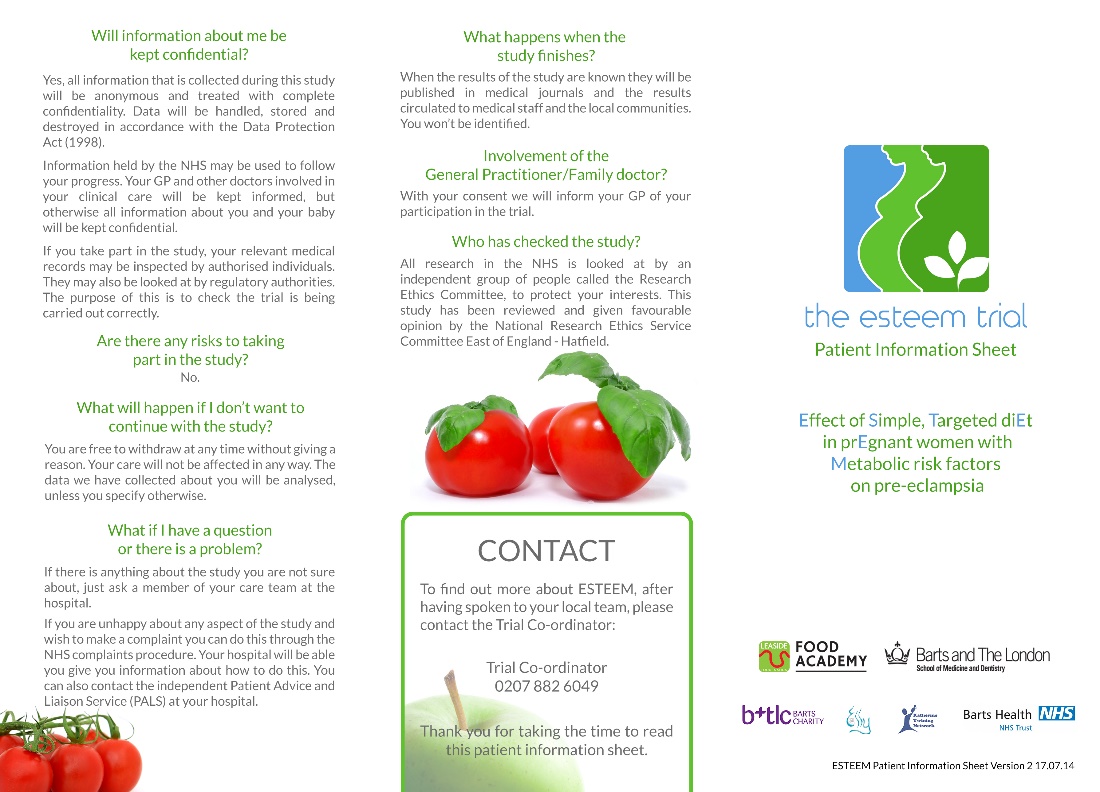


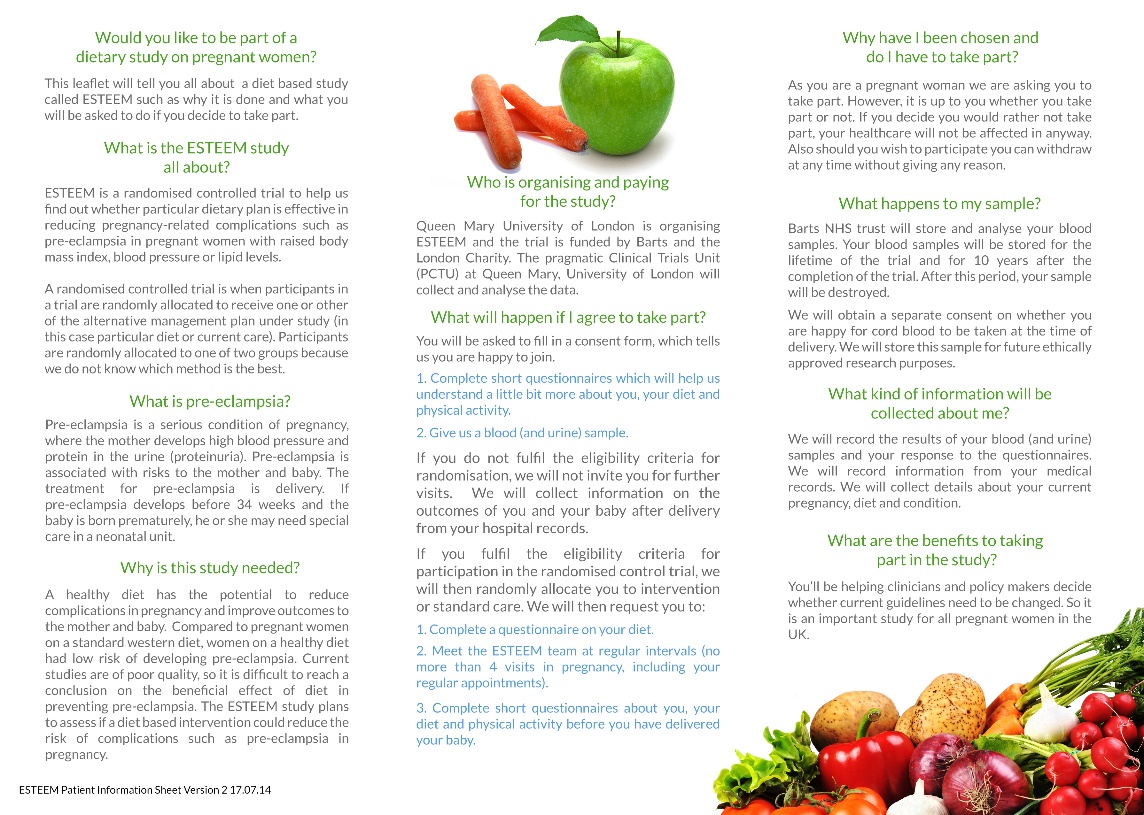

Supplement: S2 Text — ESTEEM, Effect of Simple, Targeted Diet in Pregnant Women With Metabolic Risk Factors on Pregnancy Outcomes. (DOCX) [file pmed.1002857.s005.docx]

**S4 Text:** ESTEEM bespoke recipe book

| **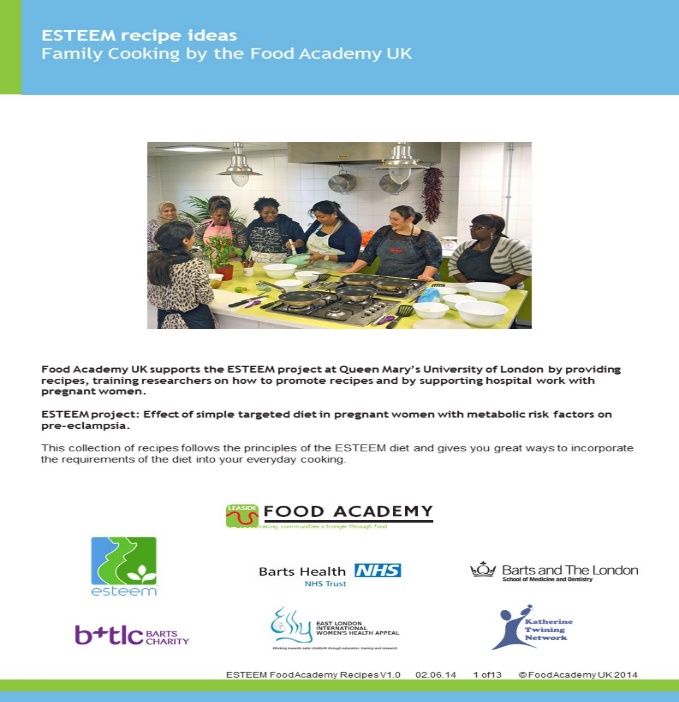** | **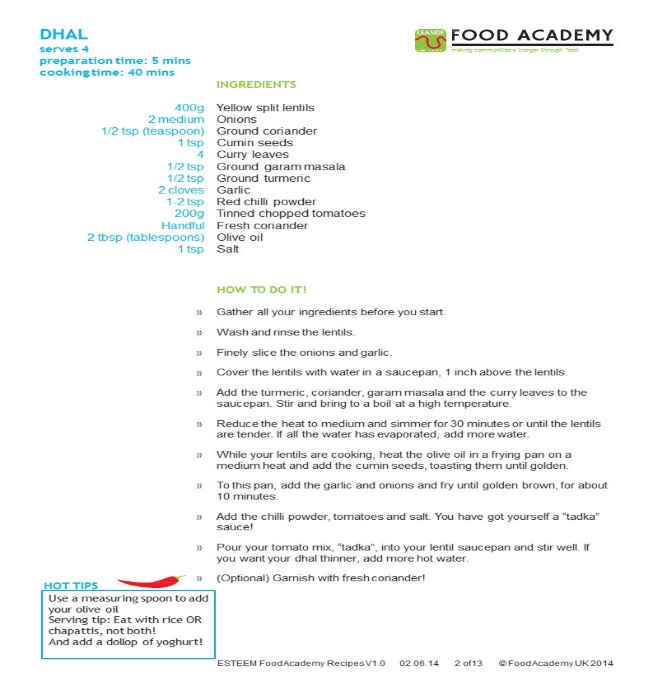** |
| --- | --- |
| **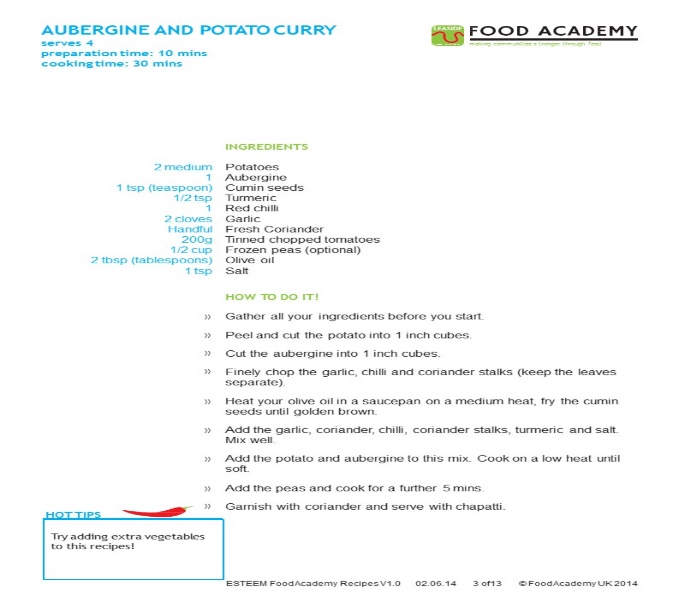** | **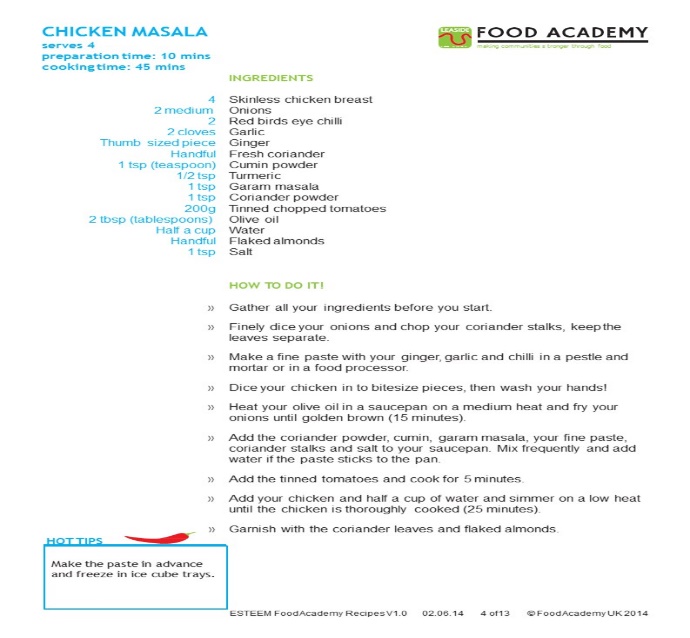** |
| **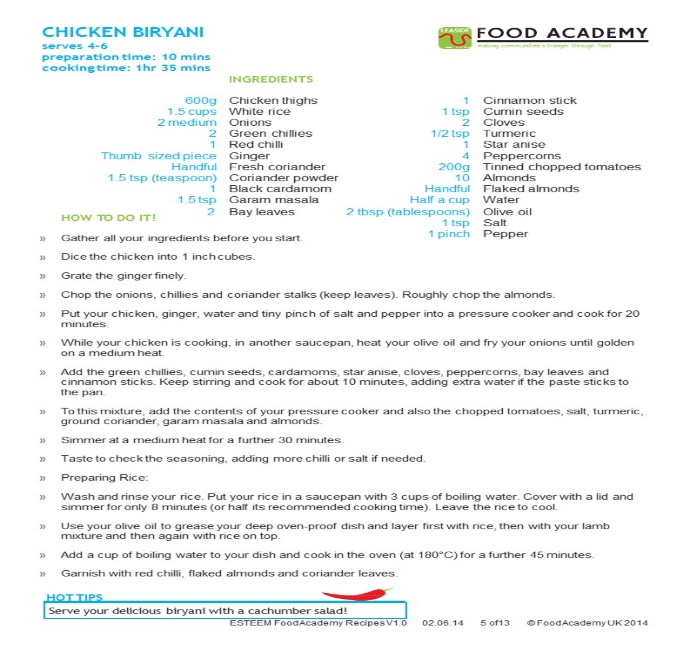** | **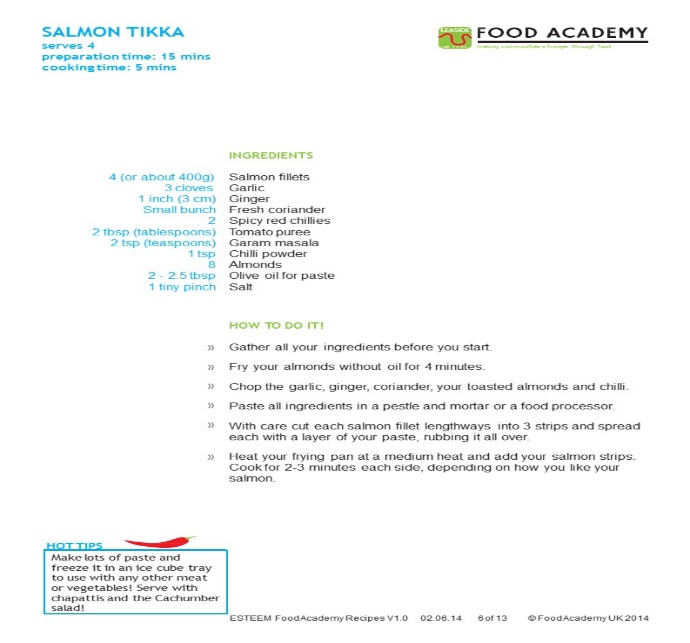** |
| **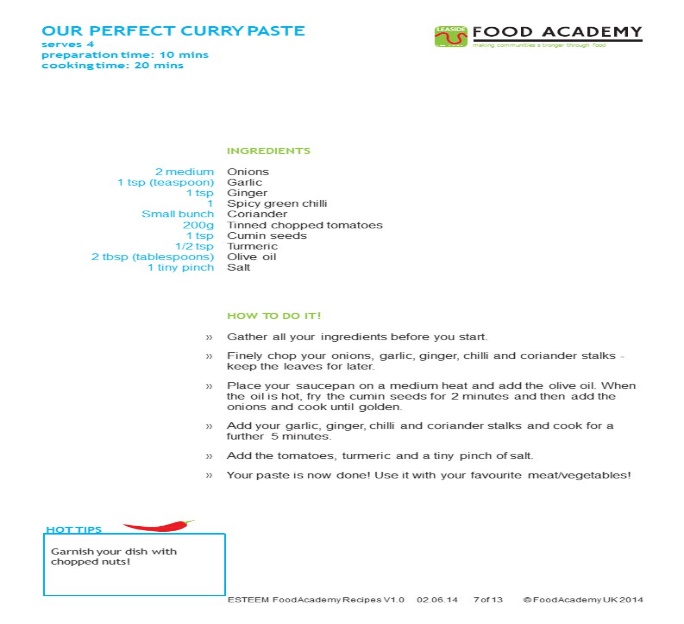** | **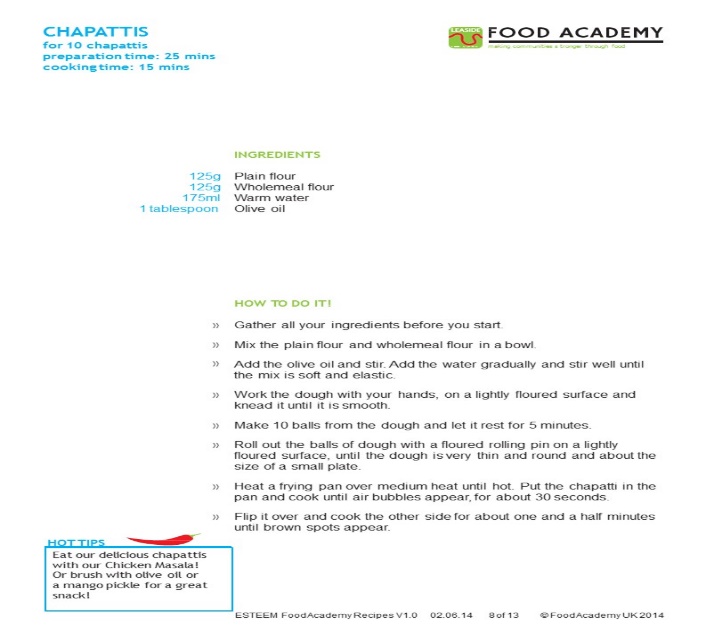** |
| **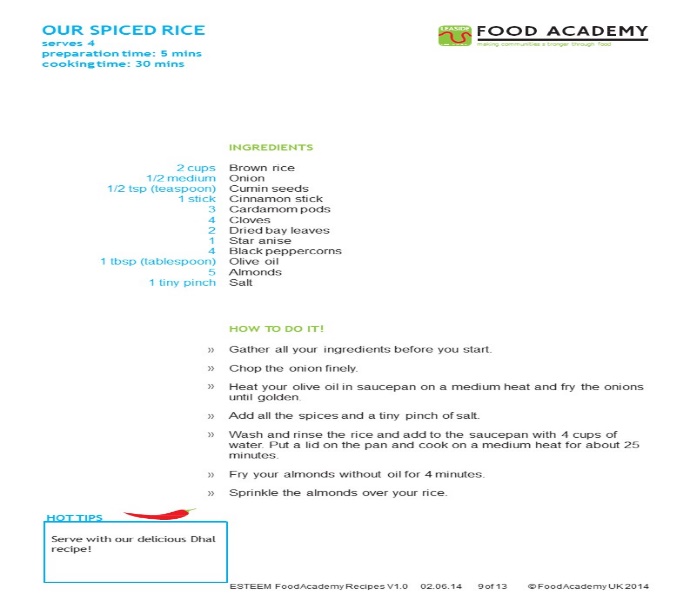** | **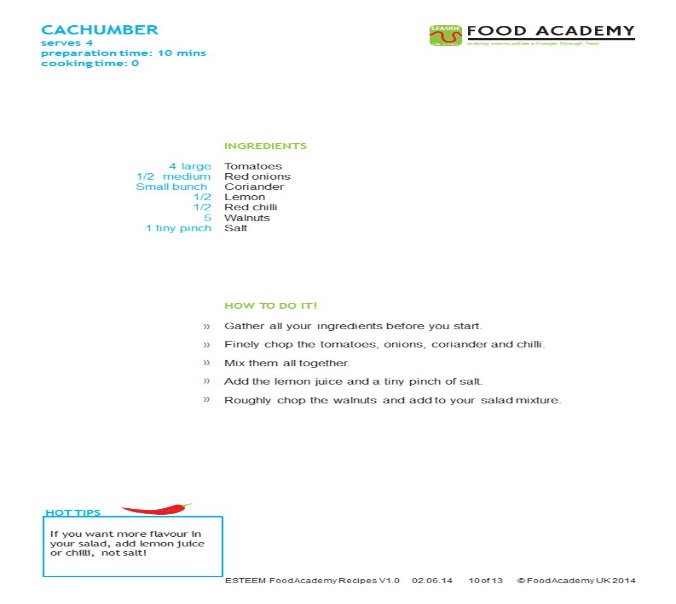** |
| **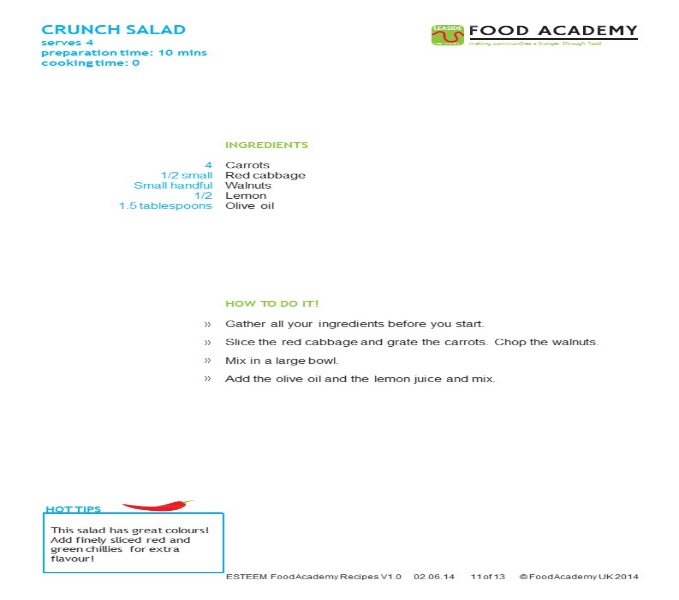** | **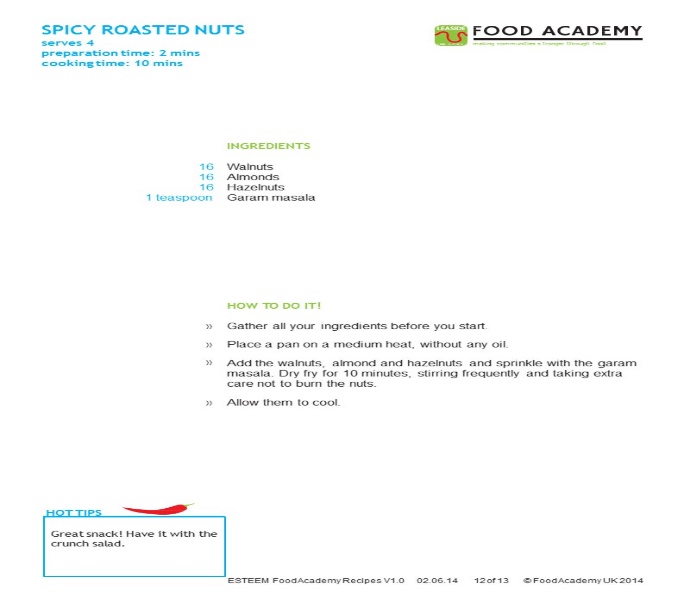** |
| **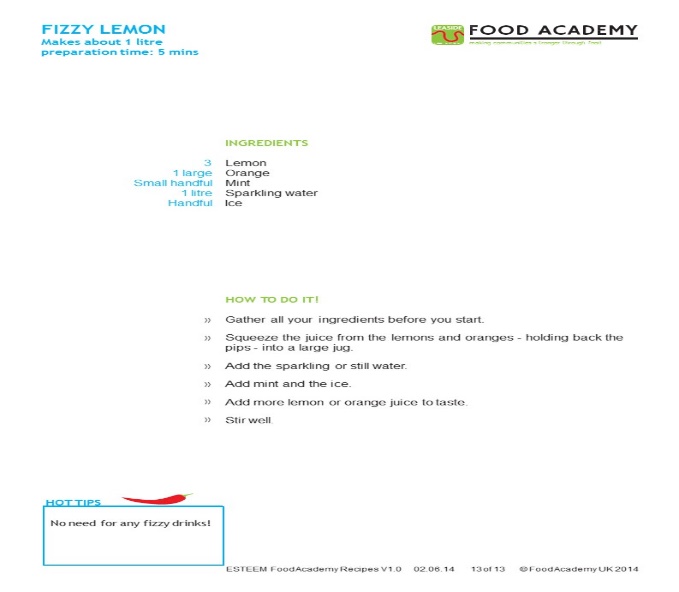** |  |

Supplement: S4 Text — ESTEEM, Effect of Simple, Targeted Diet in Pregnant Women With Metabolic Risk Factors on Pregnancy Outcomes. (DOCX) [file pmed.1002857.s007.docx]
