# Supplementary material for: Mediterranean-style diet in pregnant women with metabolic risk factors (ESTEEM): A pragmatic multicentre randomised trial
Source: PLoS Med. 2019 Jul 23;16(7):e1002857. doi: 10.1371/journal.pmed.1002857 (PMC6650045; doi:10.1371/journal.pmed.1002857)
Supplement: S3 Text — ESTEEM, Effect of Simple, Targeted Diet in Pregnant Women With Metabolic Risk Factors on Pregnancy Outcomes. (DOCX) [file pmed.1002857.s006.docx]

**S3 Text:** Intervention facts-sheets and educational presentation for the ESTEEM trial

3A: General information


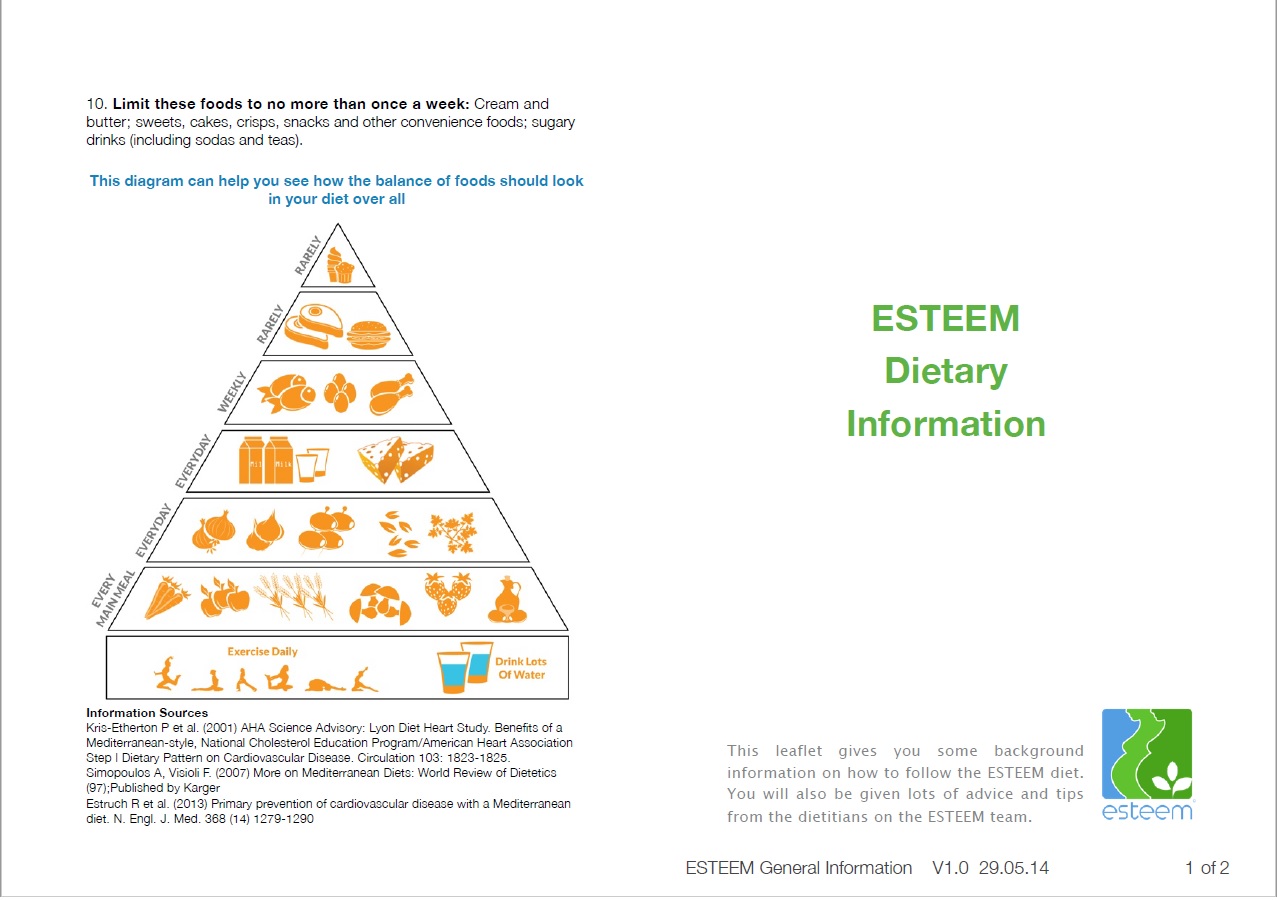


**
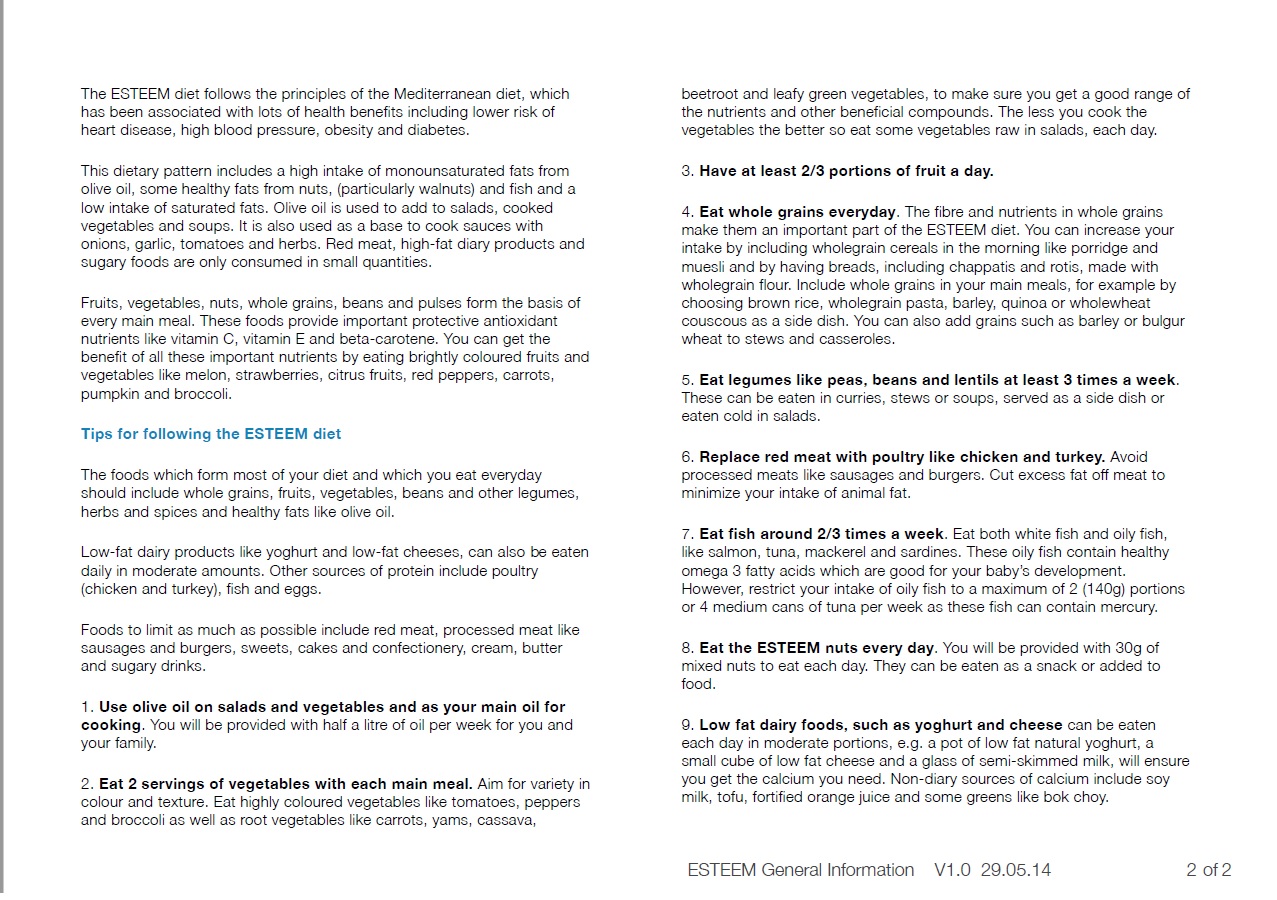
**

3B: Weekly plan for individual food portion sizes


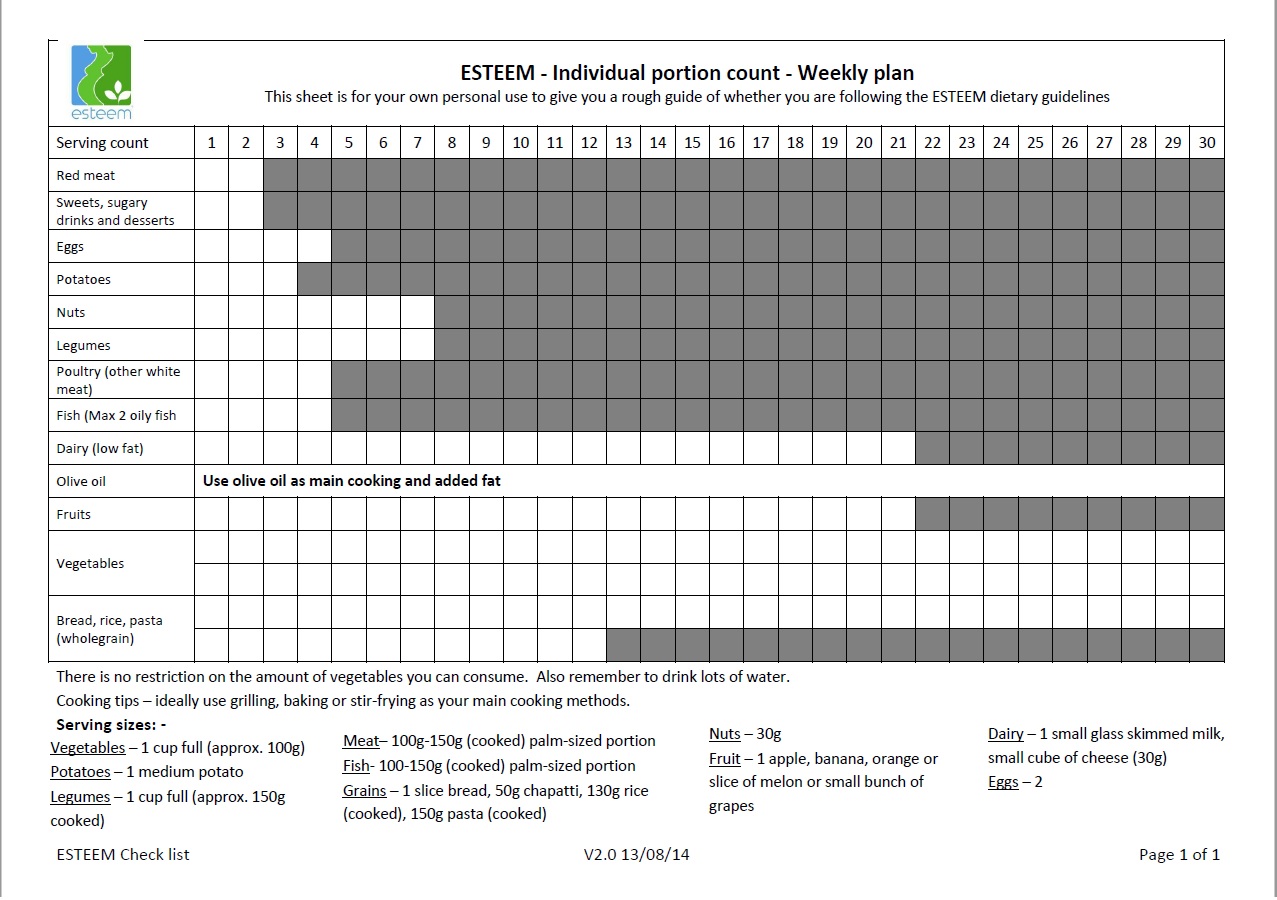


3C: Leaflet on extra virgin olive oil


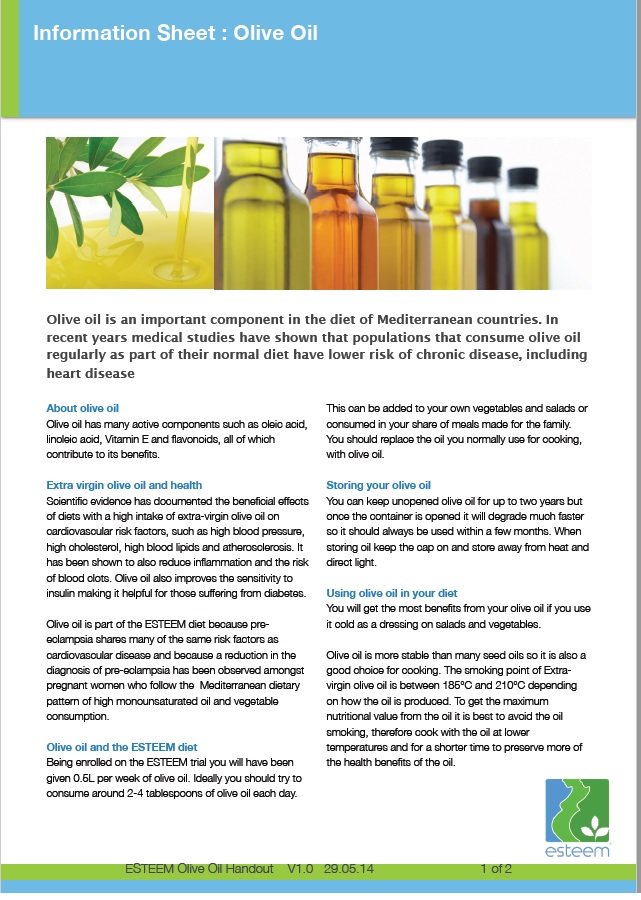

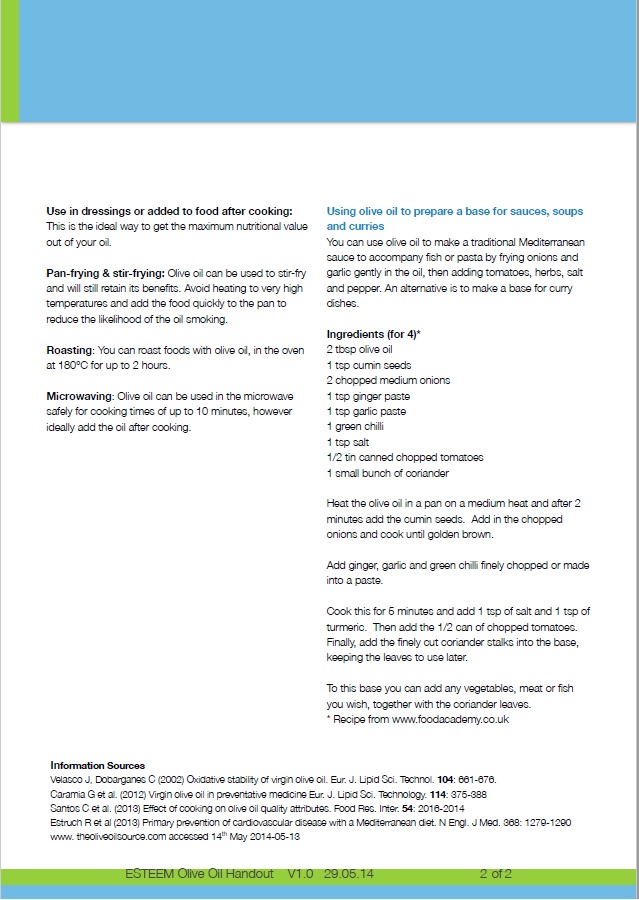


3D: Leaflet on mixed nuts


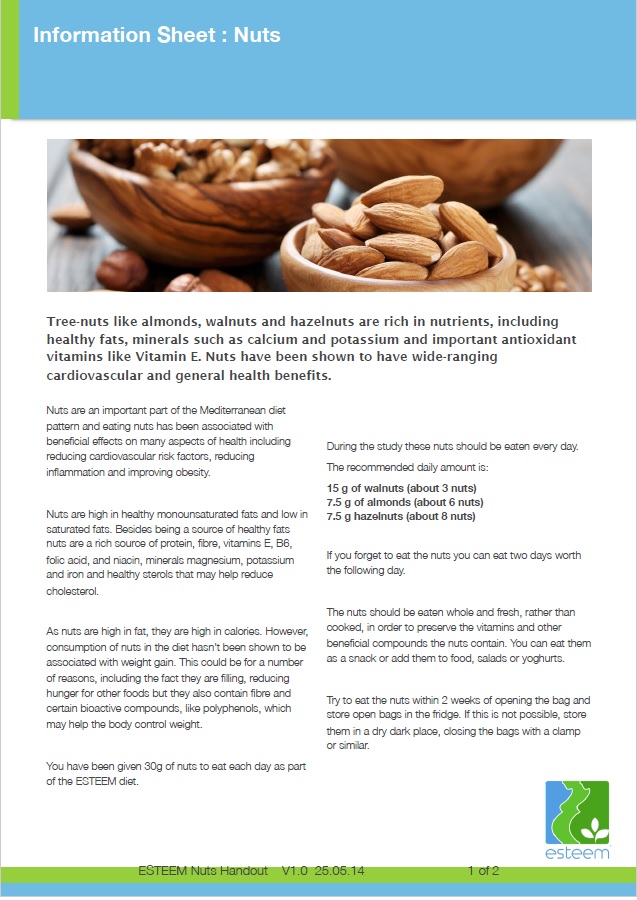

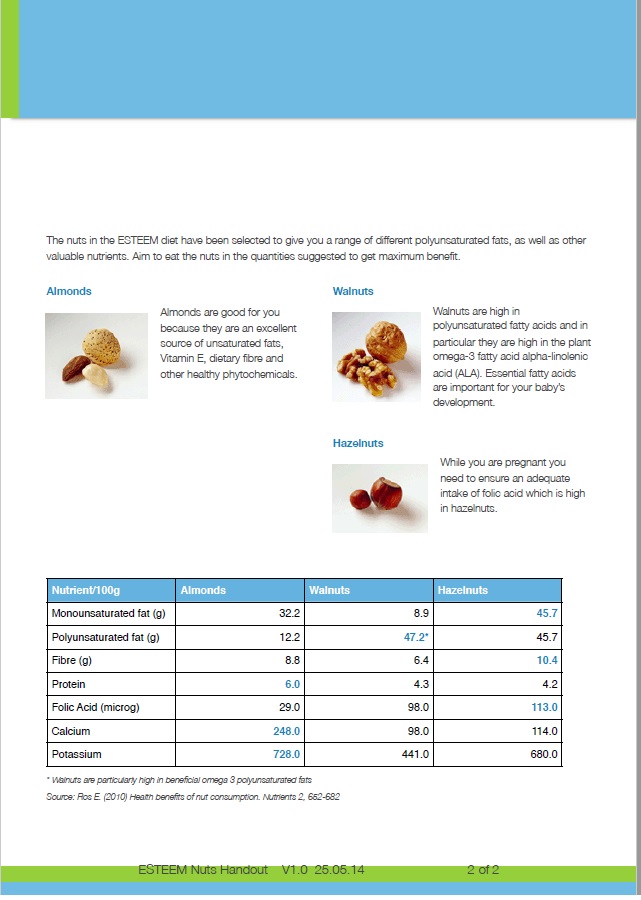


3E: Standardized educational presentation

(i) At 20 weeks’ gestation

| 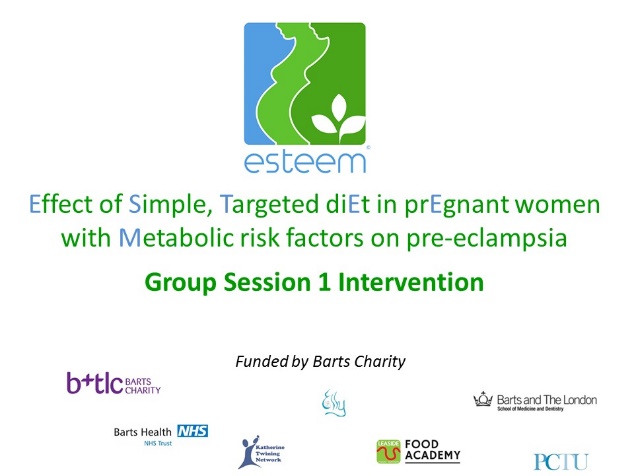 | 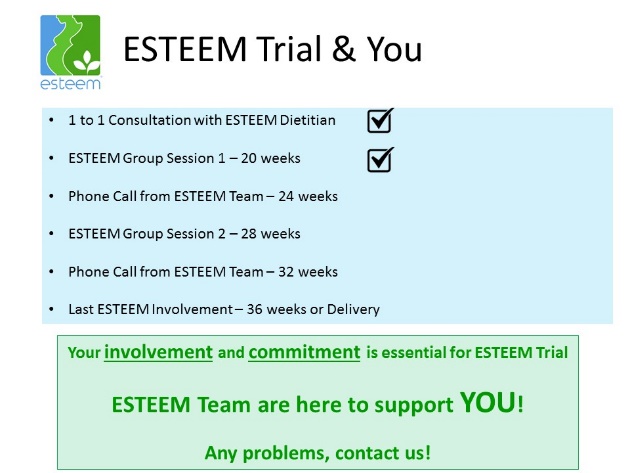 |
| --- | --- |
| 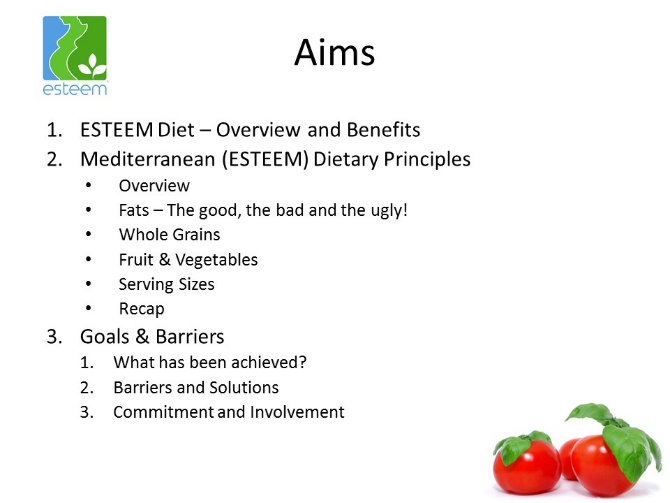 | 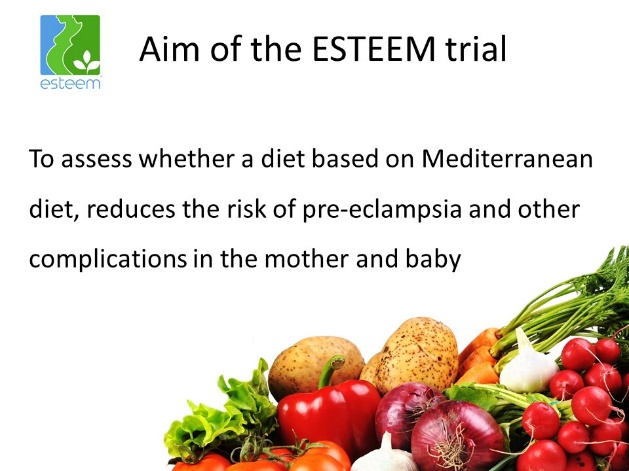 |
| 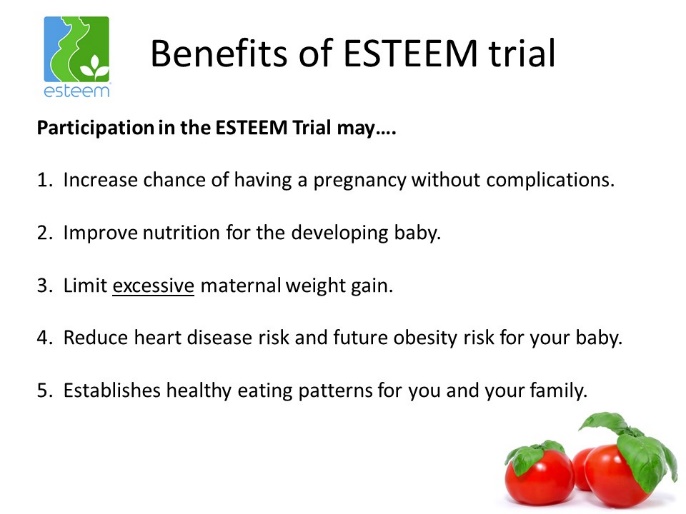 | 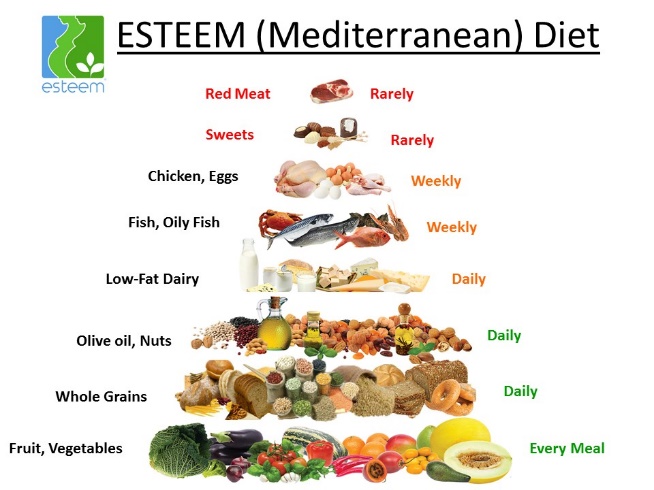 |
| 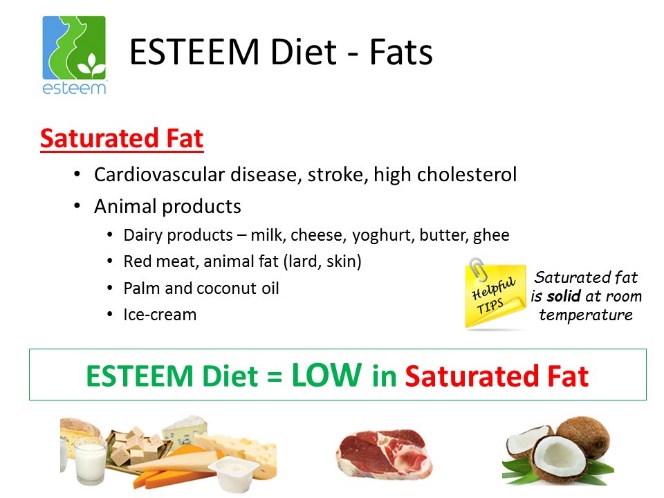 | 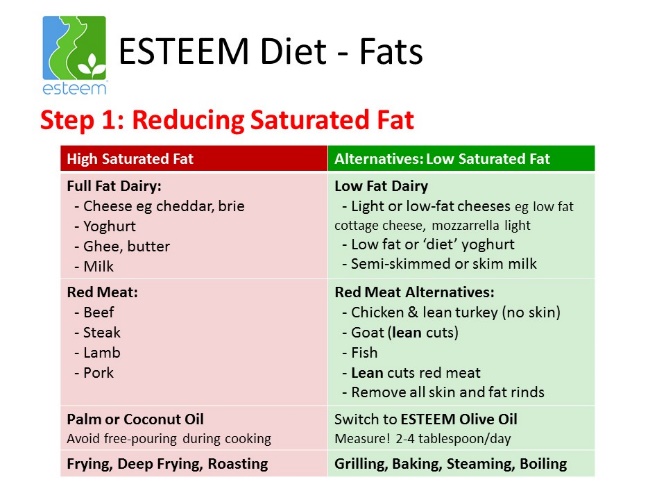 |
| 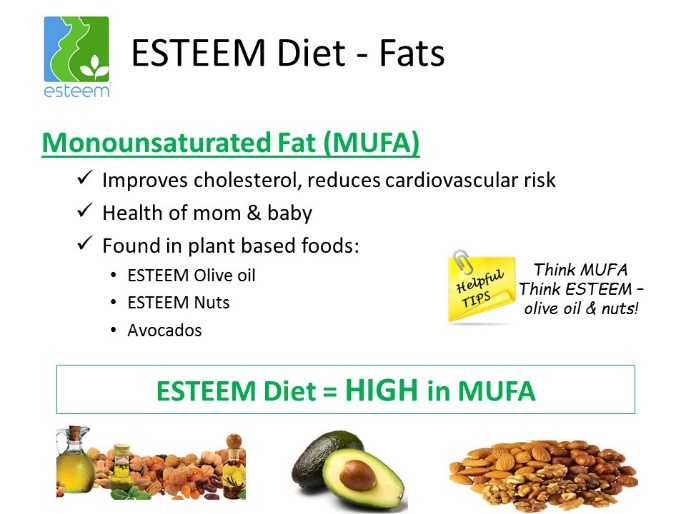 | 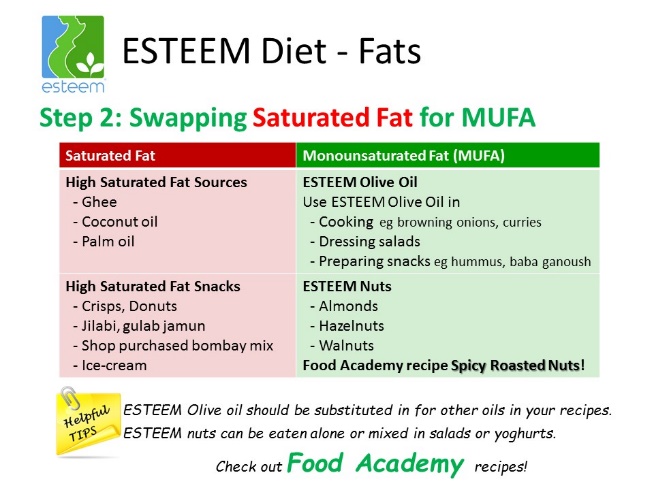 |
| 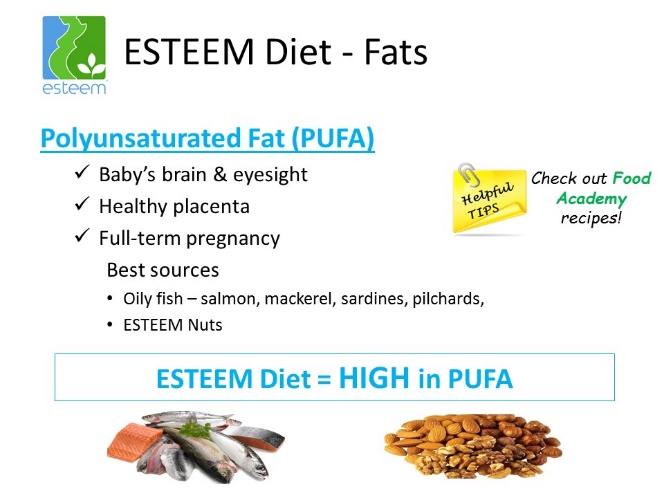 | 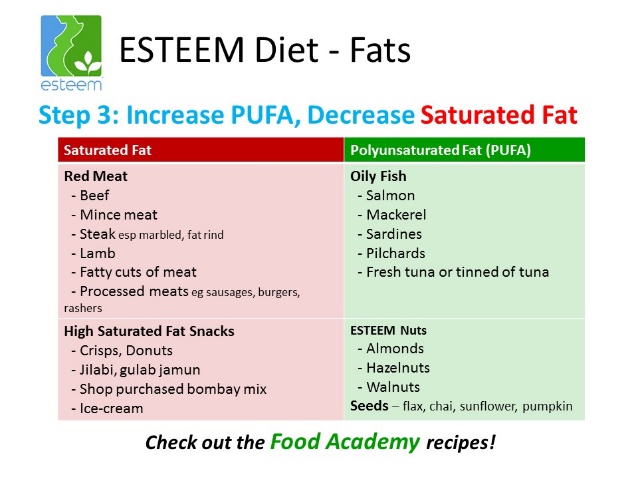 |
| 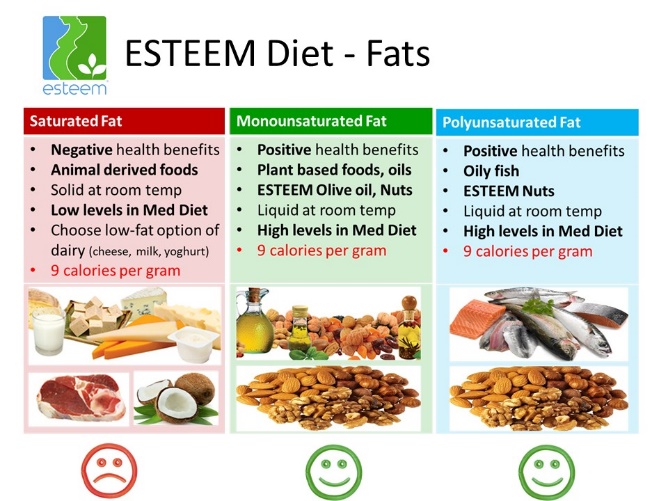 | 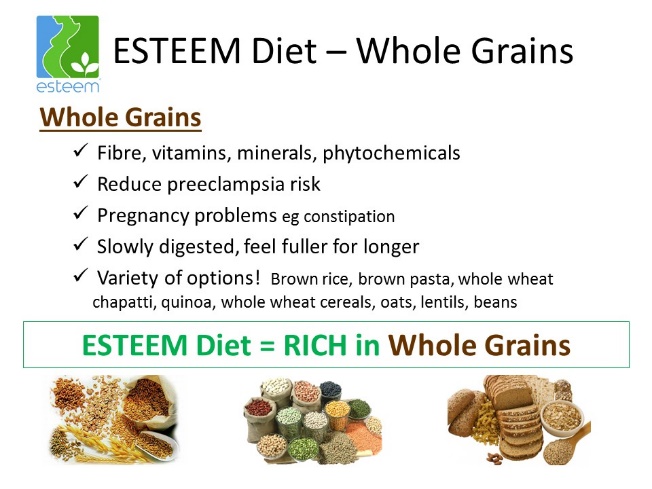 |
| 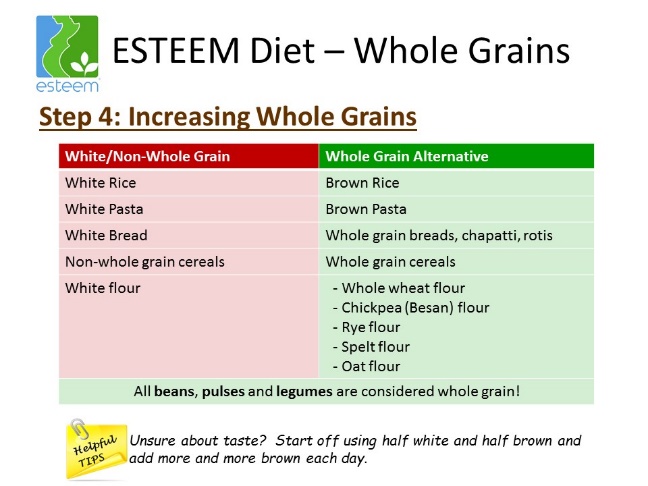 | 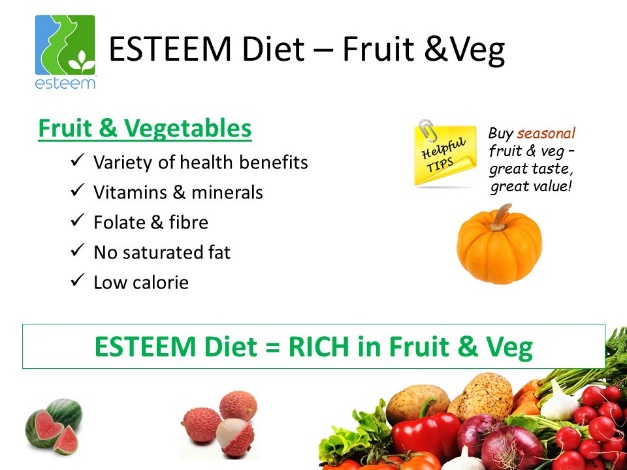 |
| 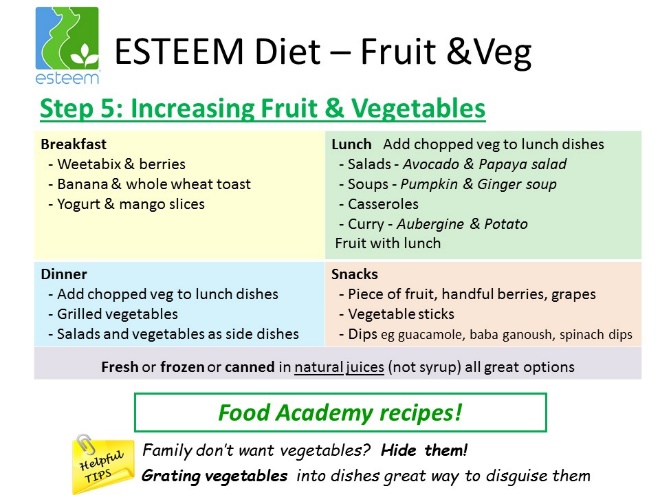 | 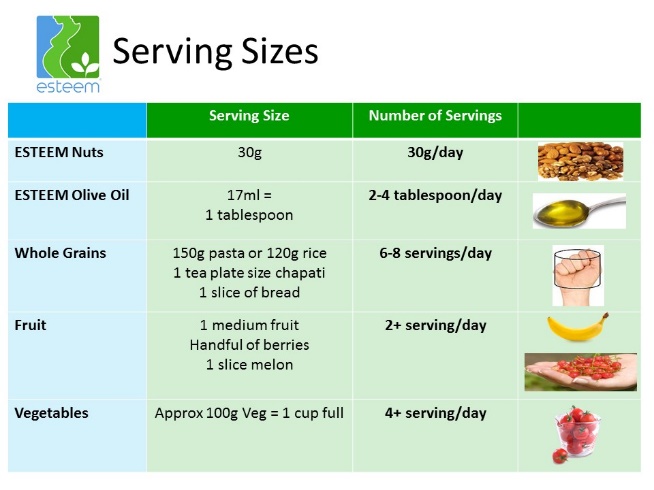 |
| 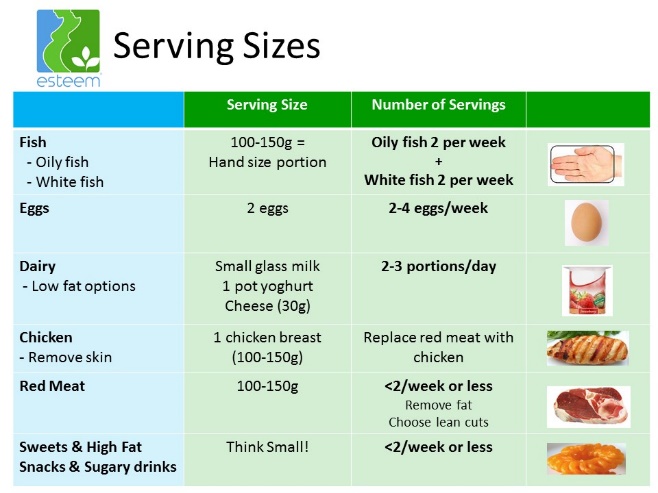 | 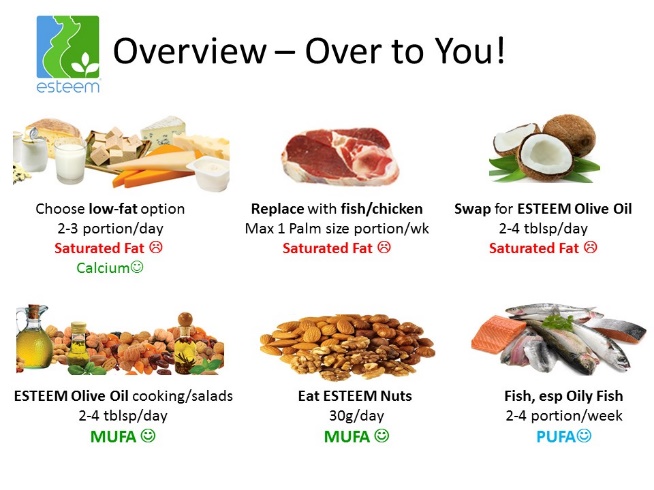 |
| 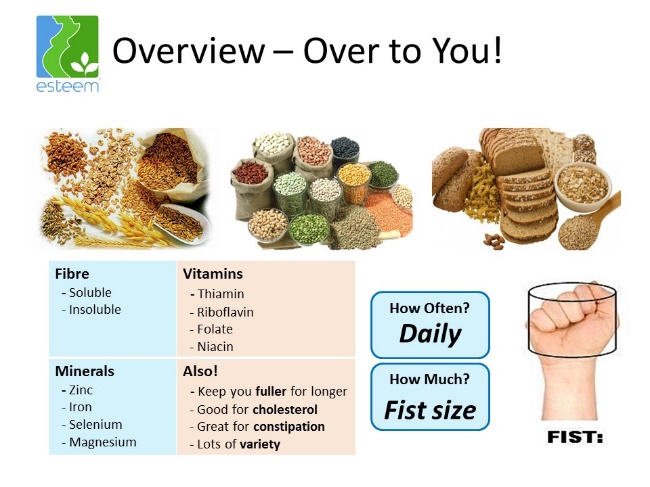 | 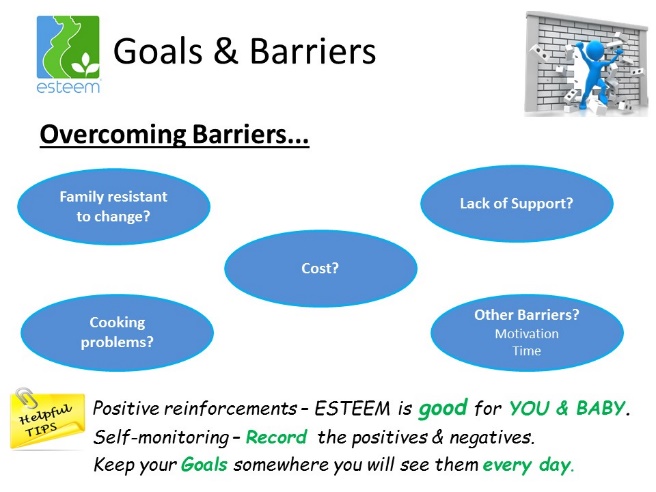 |
| 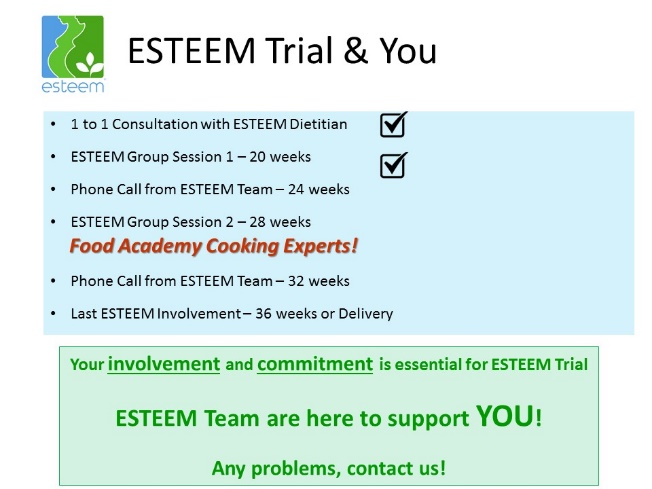 | 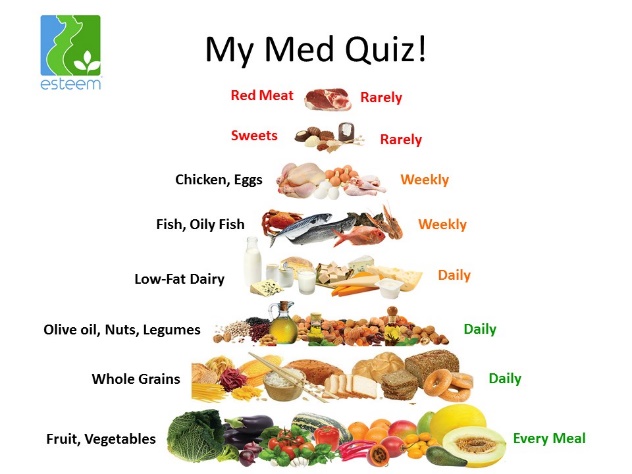 |
| 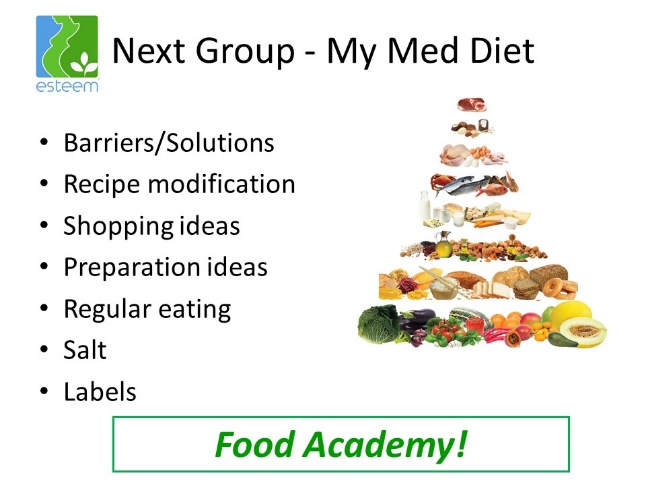 | 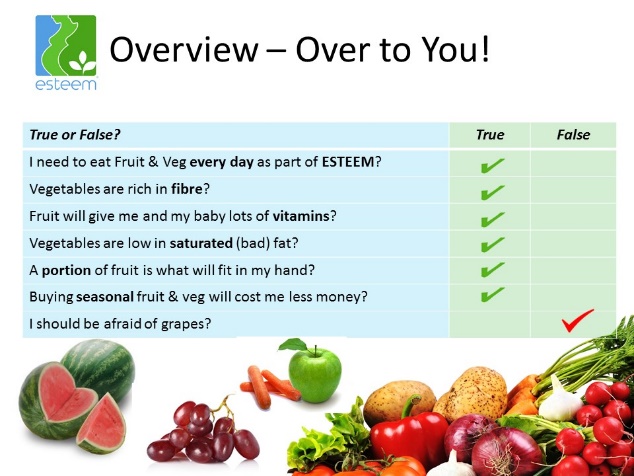 |

(ii) At 28 weeks’ gestation

| 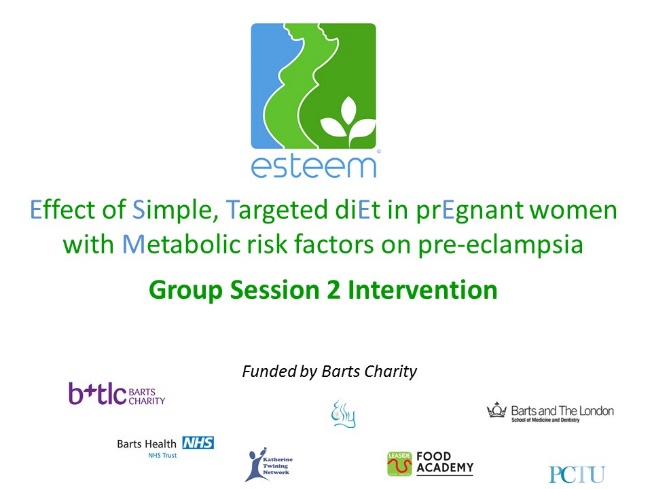 | 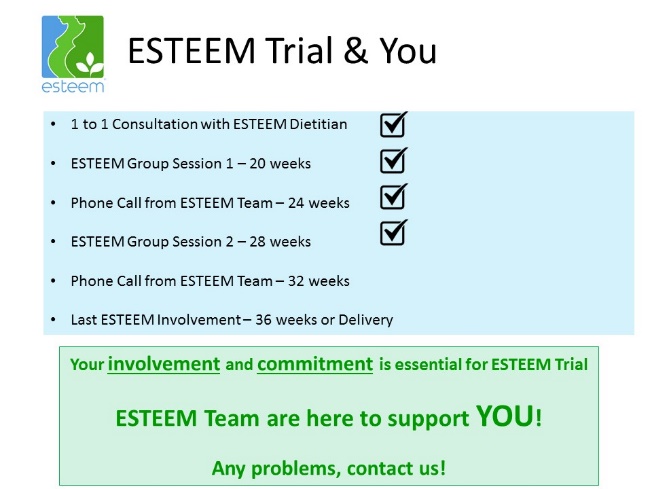 |
| --- | --- |
| 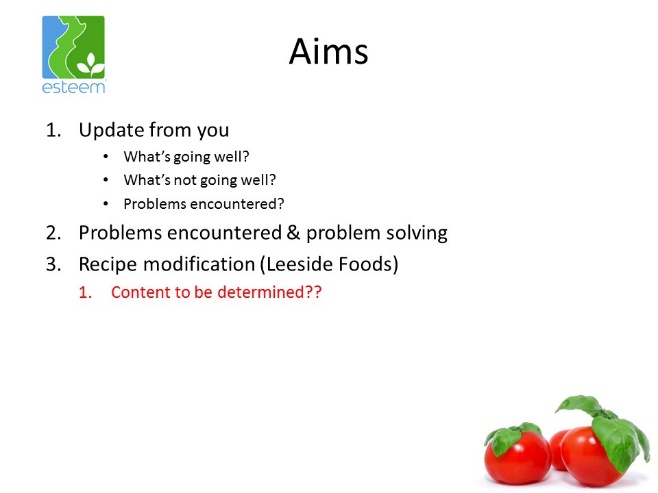 | 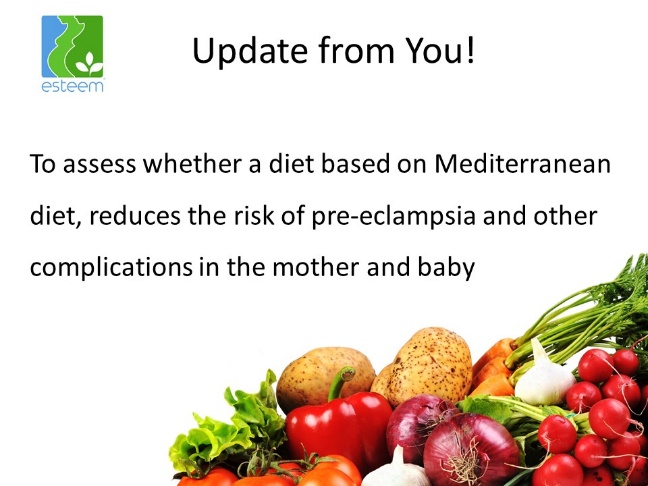 |
| 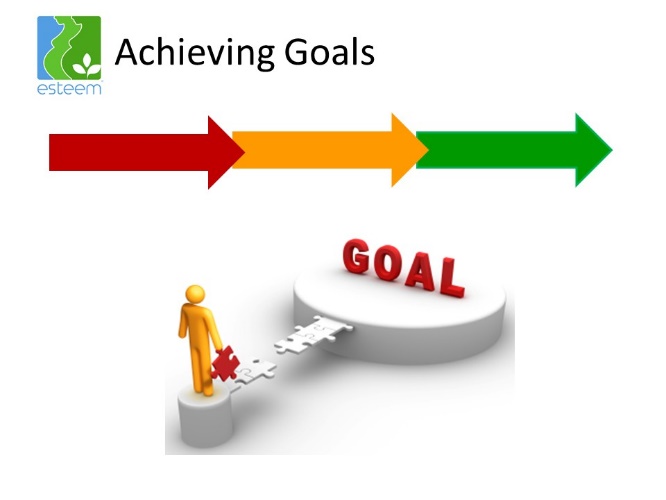 | 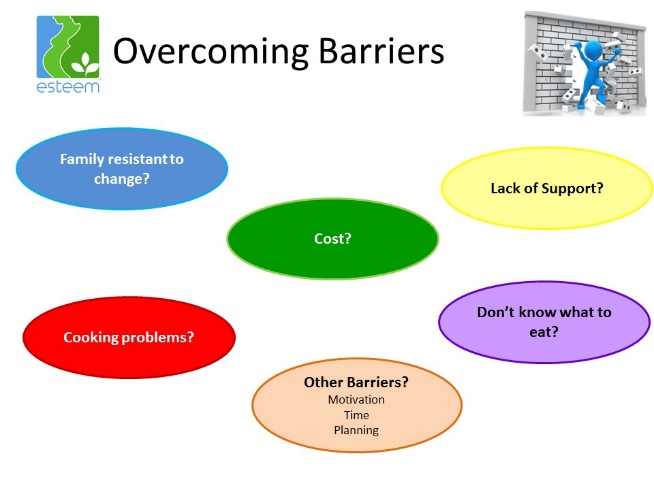 |
| 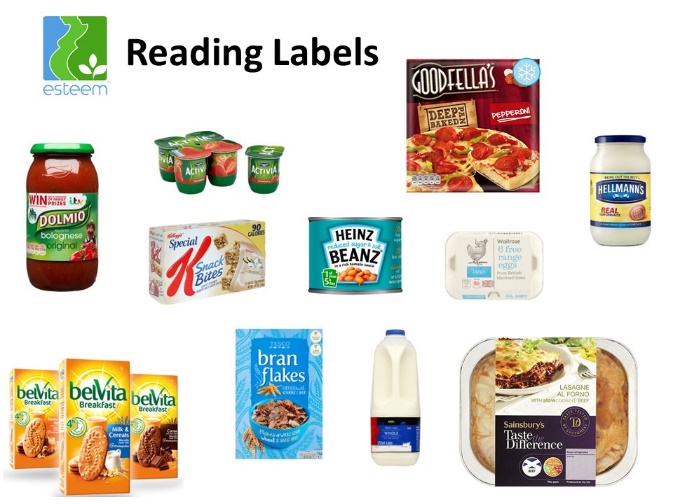 | 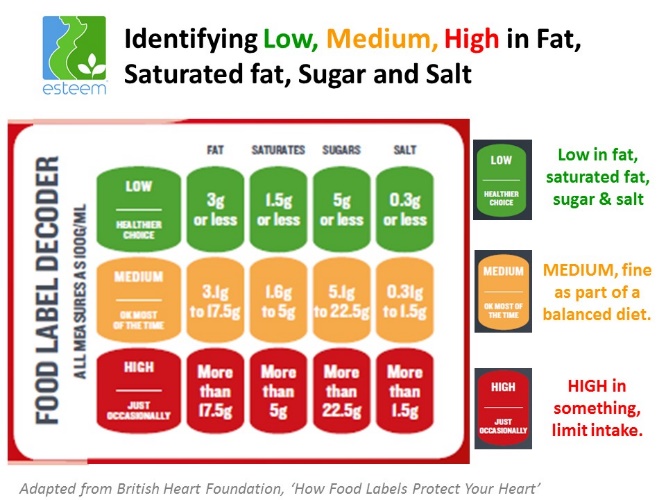 |
| 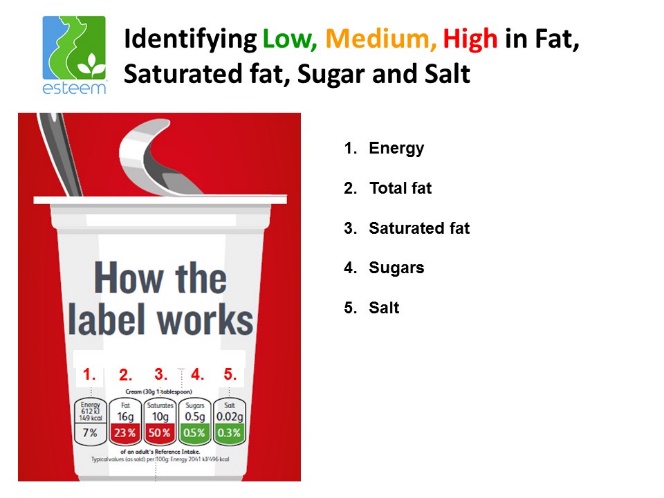 | 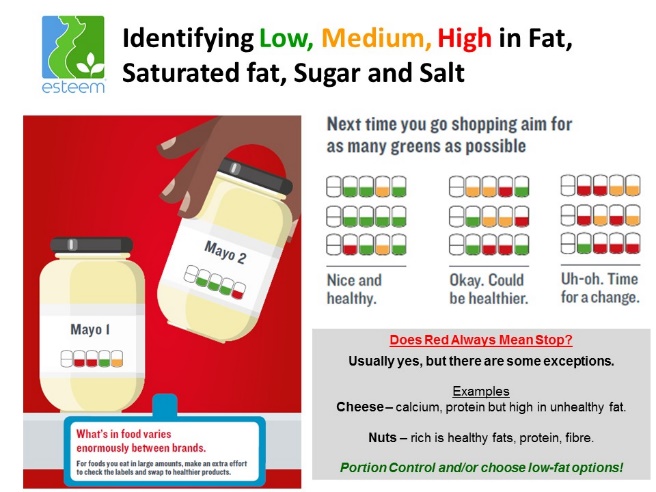 |
| 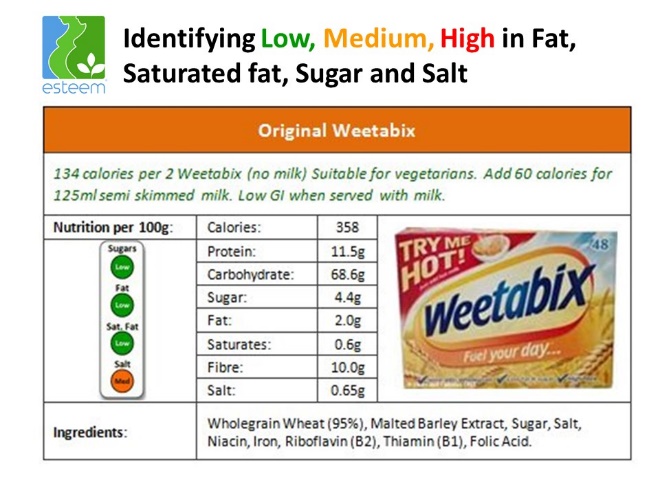 | 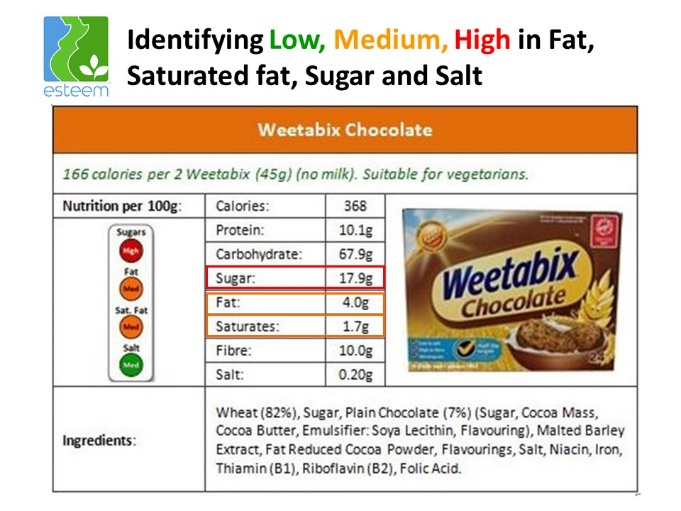 |
| 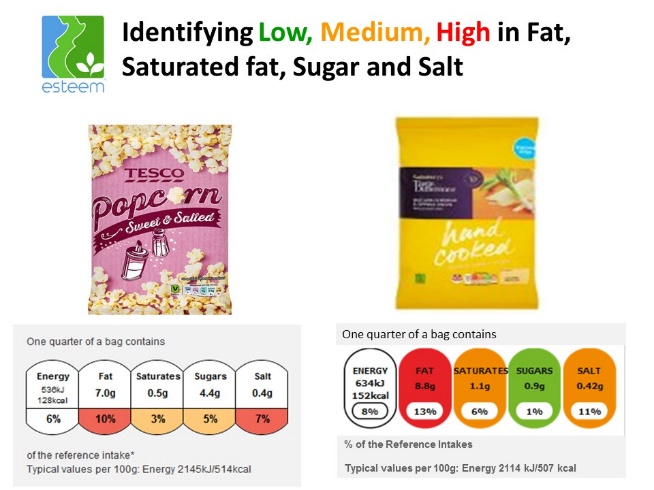 | 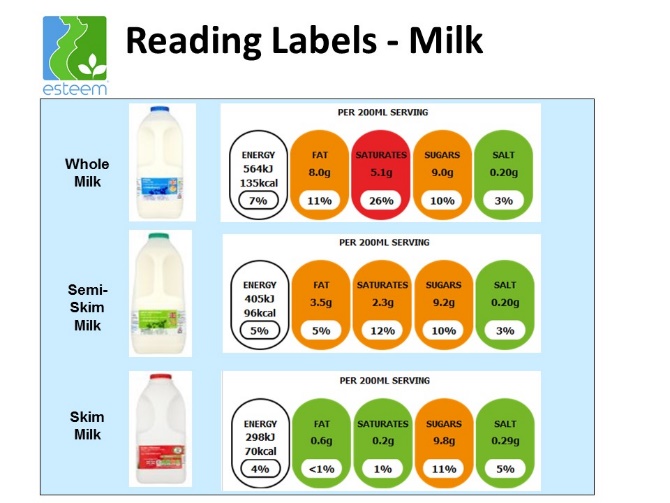 |
| 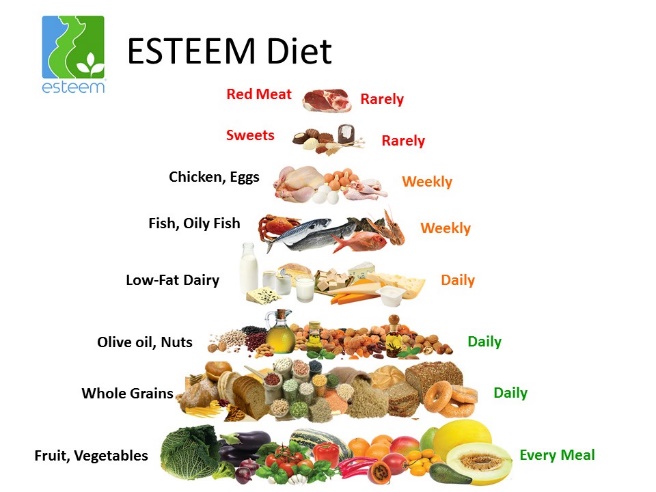 | 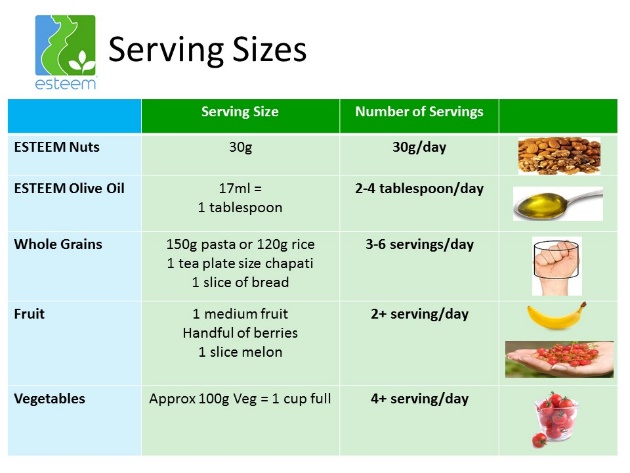 |
| 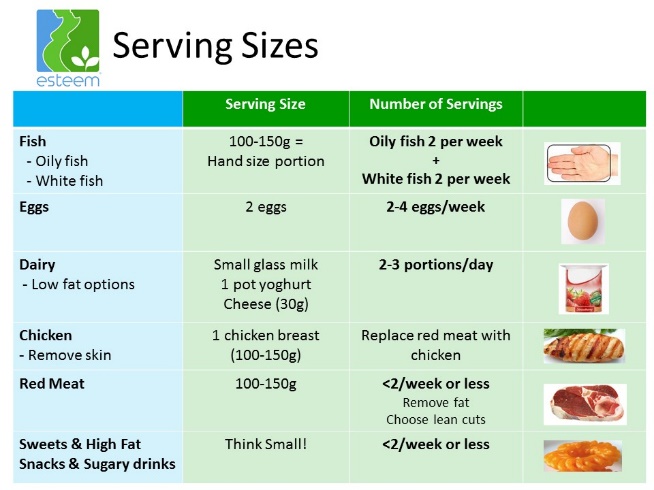 |  |

3F: Composition of daily nuts and extra virgin oil intake in the intervention

- *0∙5 litre/week extra virgin olive oil:*

Each 100g provides:

| Protein | Trace |
| --- | --- |
| Carbohydrates | 0.0g |
| Fat | 99.9g |
| Saturated fat | 14.0g |
| Mono-unsaturated fat | 69.7g |
| Poly-unsaturated fat | 11.2g |
| Fibre | 0.0g |
| Sodium | Trace |

- *30 g of mixed nuts:*

15 g of walnuts (about 3 nuts)

7.5 g of almonds (about 6 nuts)

7.5 g hazelnuts (about 8 nuts**)**
