# Supplementary material for: Mediterranean-style diet in pregnant women with metabolic risk factors (ESTEEM): A pragmatic multicentre randomised trial
Source: PLoS Med. 2019 Jul 23;16(7):e1002857. doi: 10.1371/journal.pmed.1002857 (PMC6650045; doi:10.1371/journal.pmed.1002857)

**S5 Text:** ESTEEM Q, EQ5D, IPAQ, FFQ, and 24 hour recall questionnaires

5A: ESTEEM Q

**
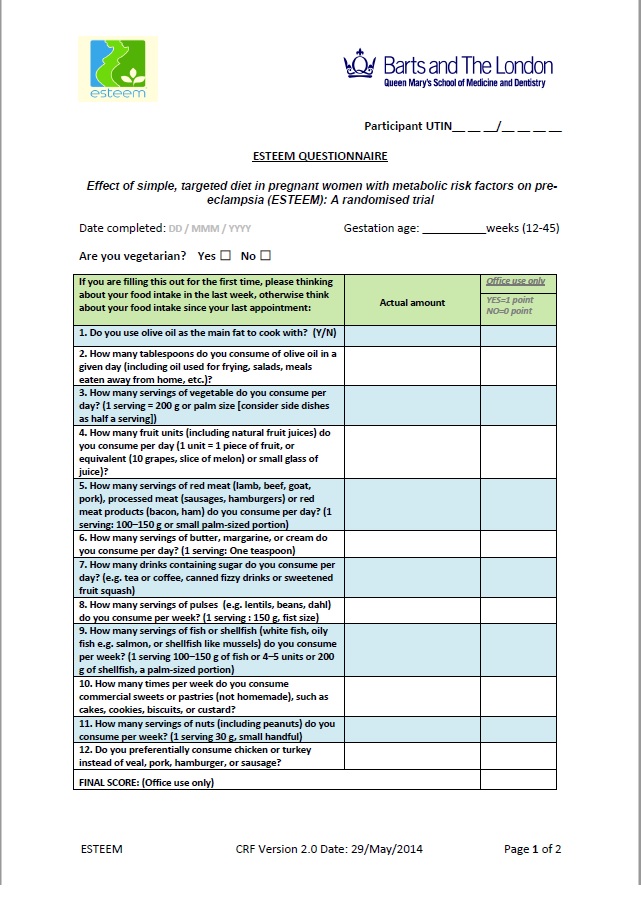

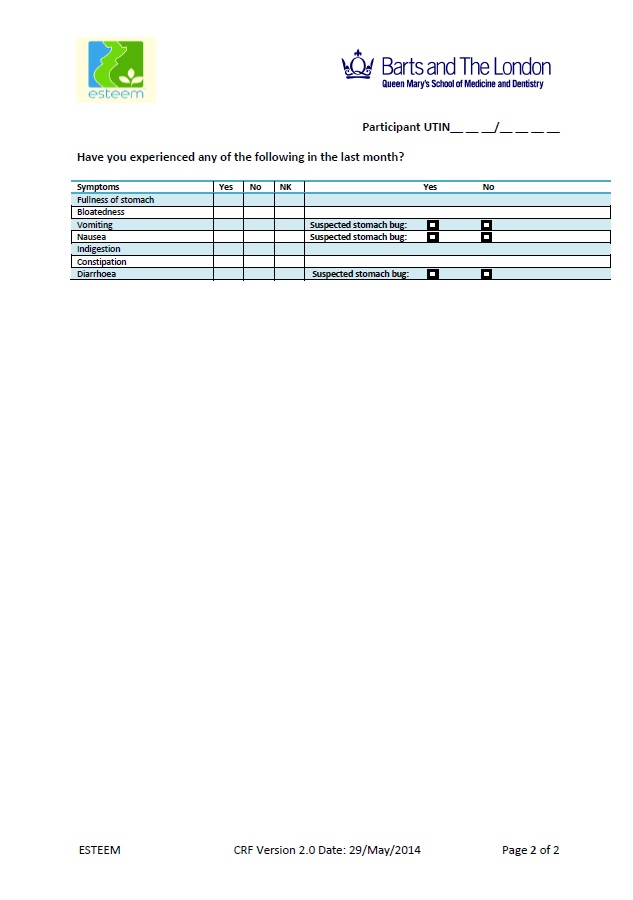
**

5B: EQ-5D

**
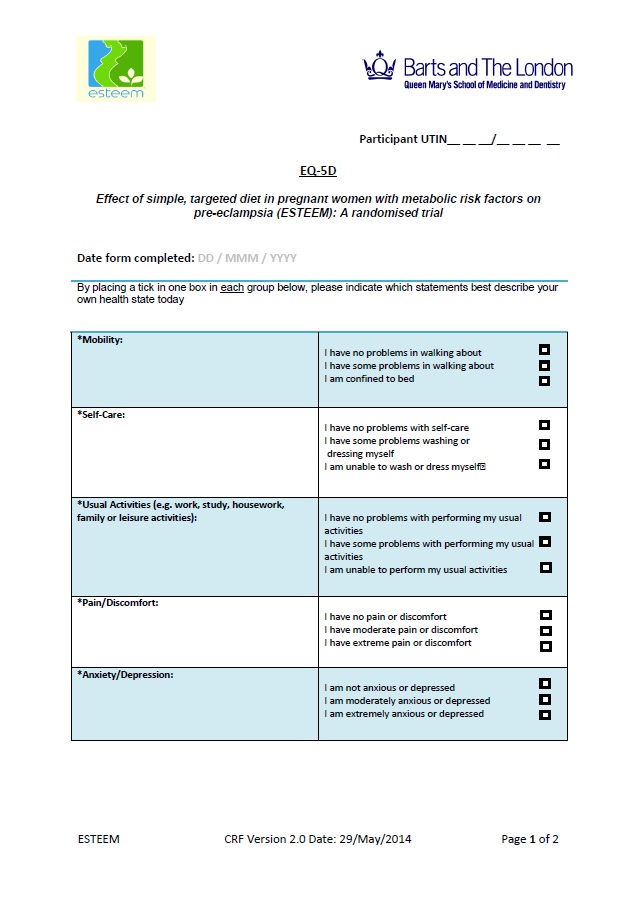

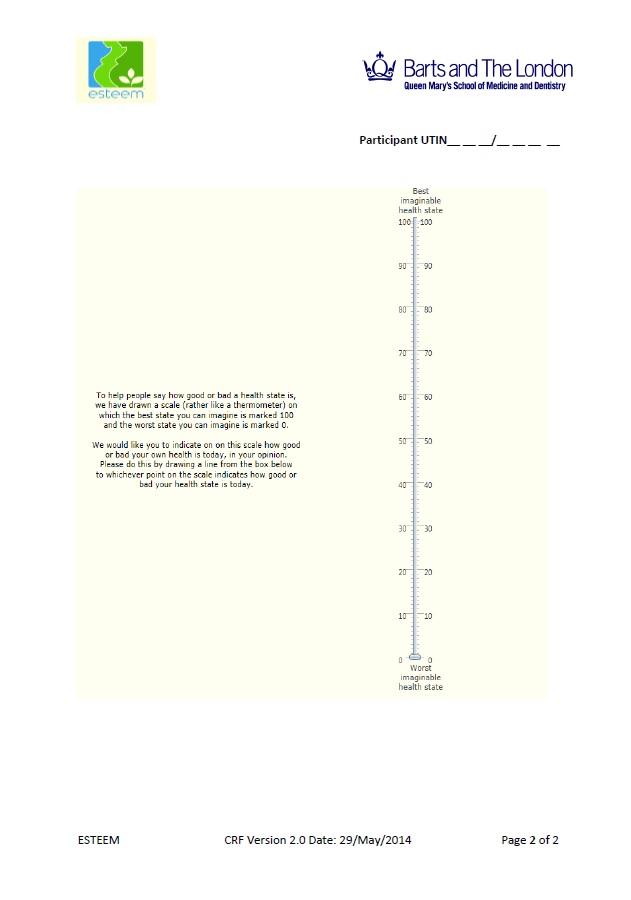
**

5C: IPAQ for physical activity

**
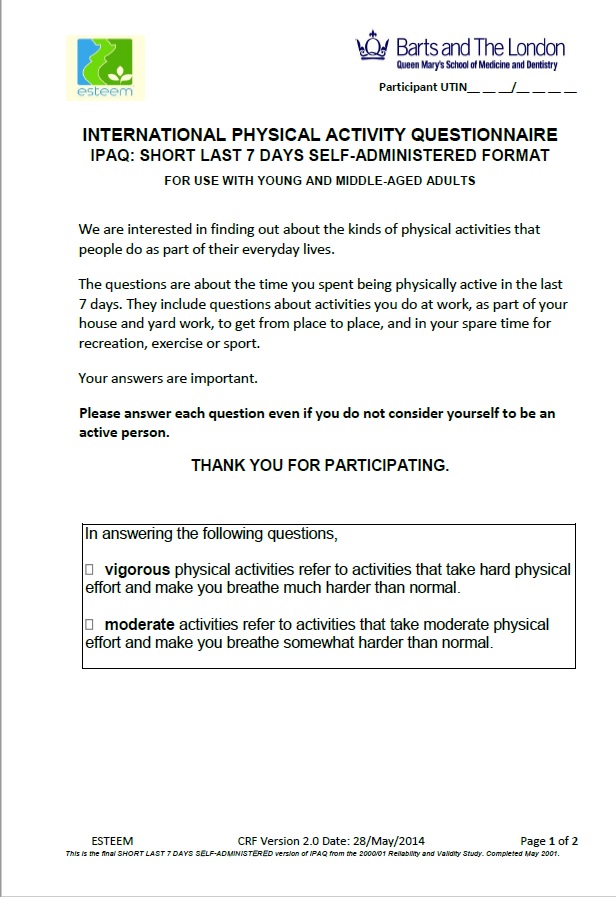

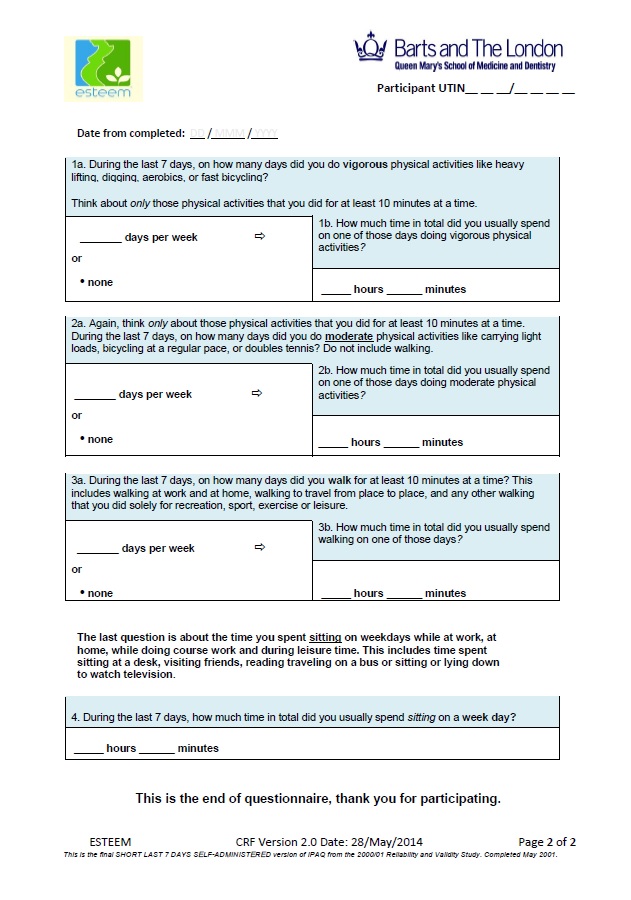
**

5D: Food Frequency Questionnaire (FFQ) for dietary intake

**
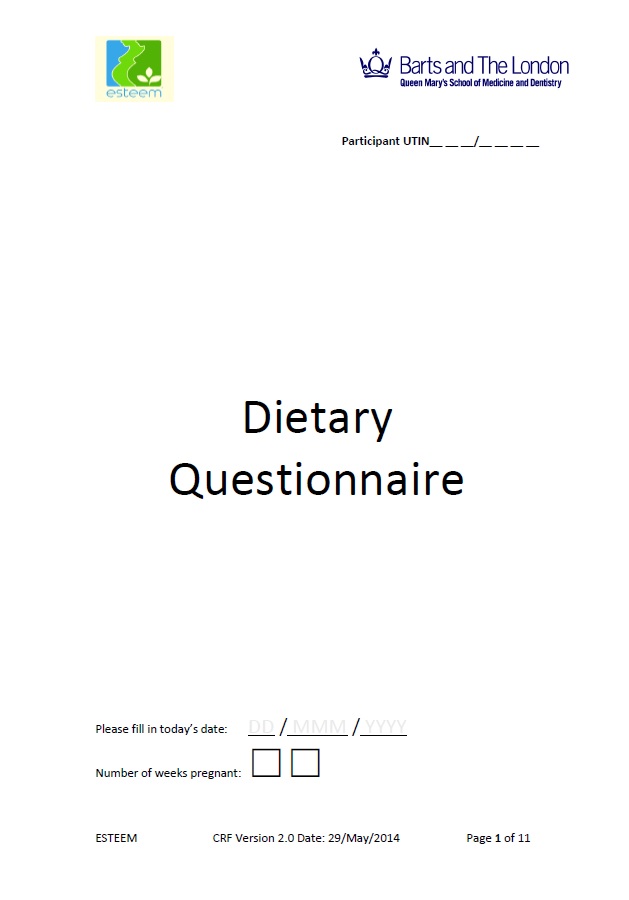

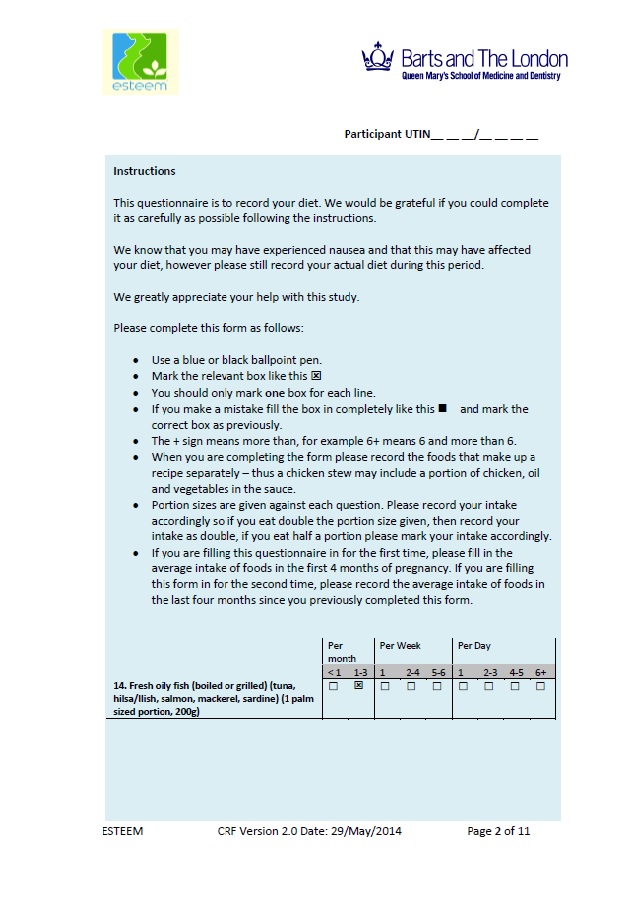
**

**
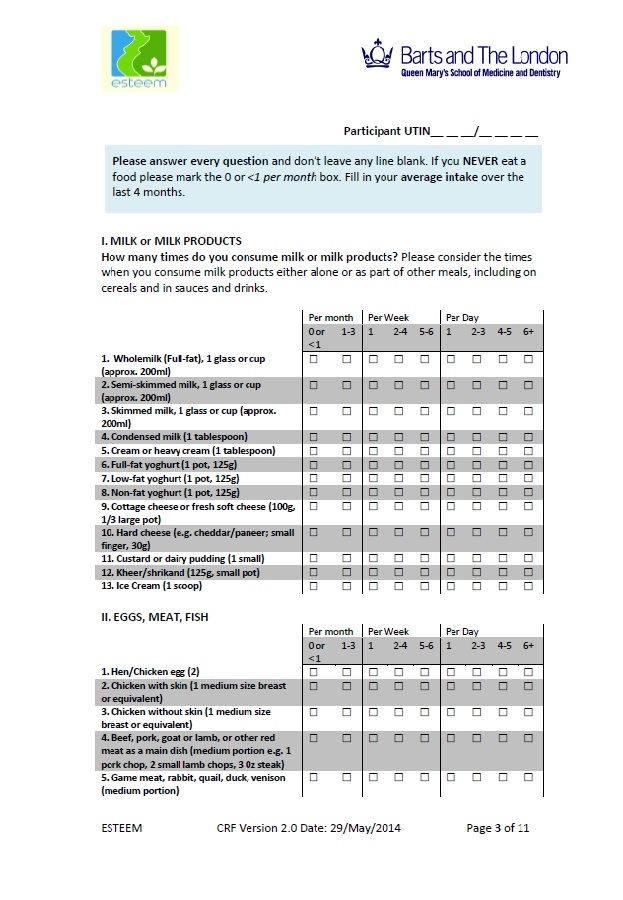

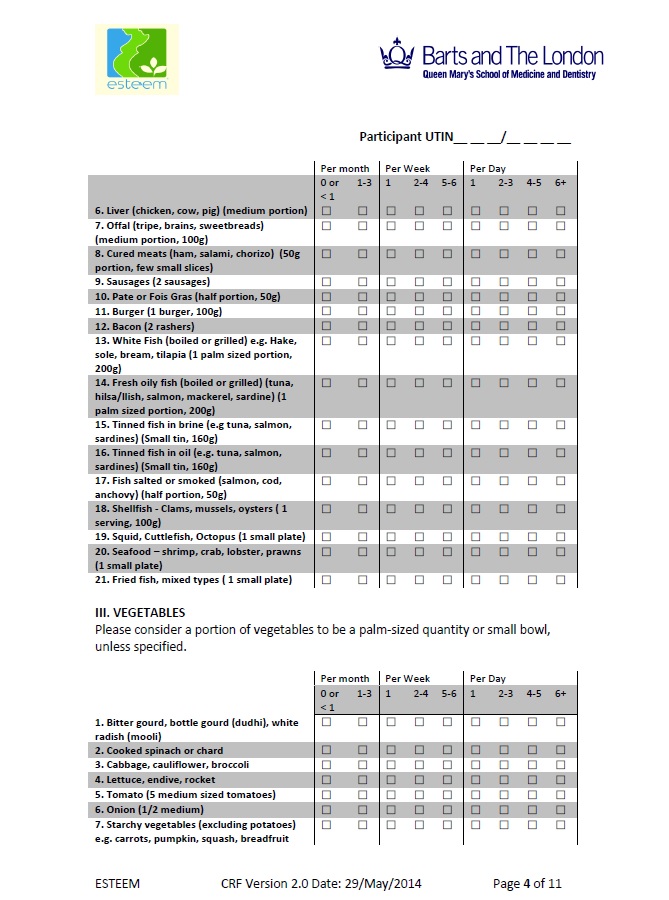
**

**
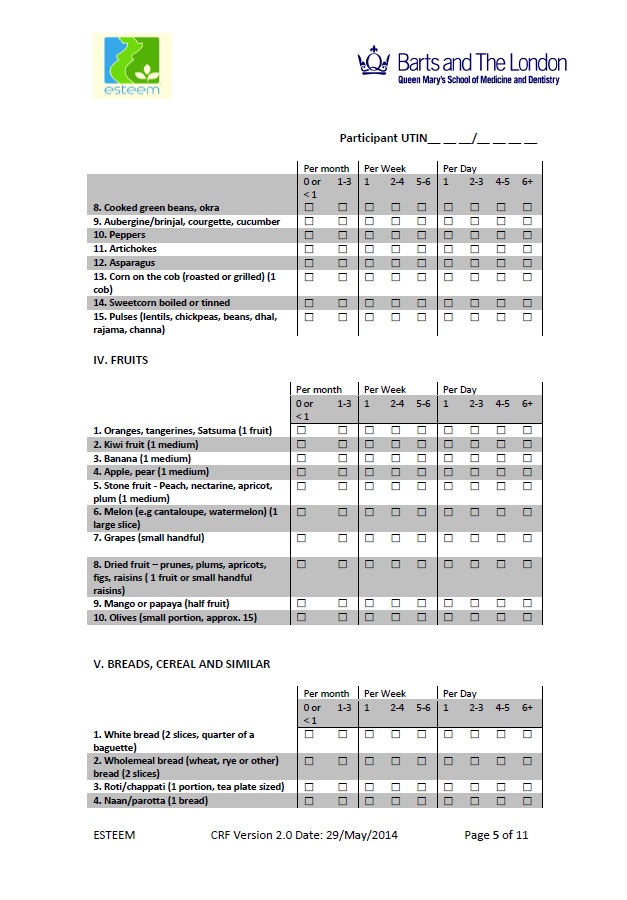

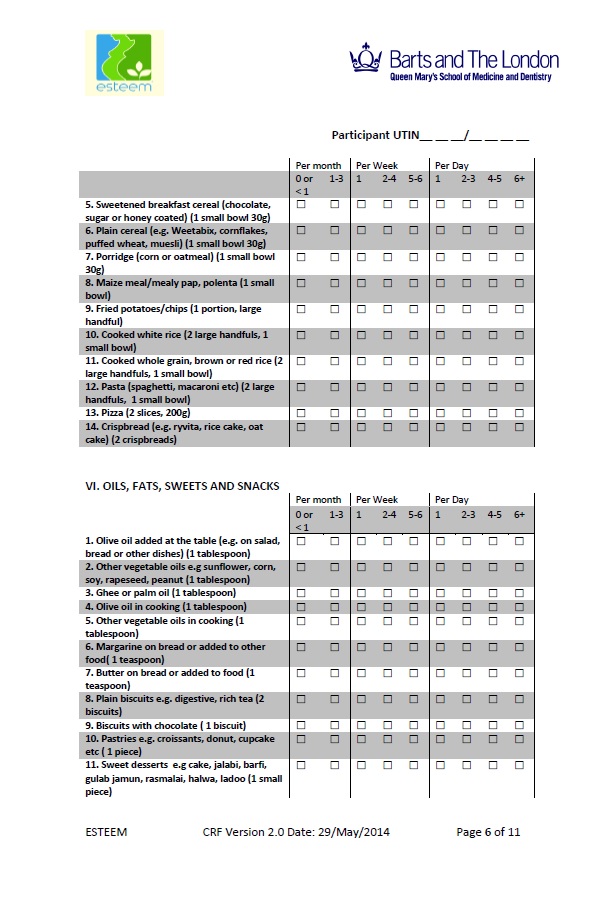
**

**
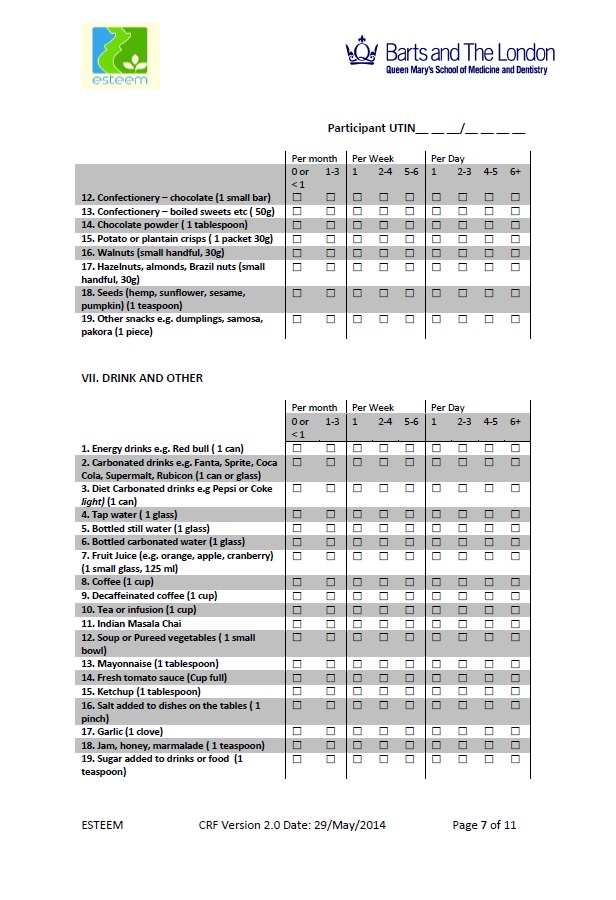

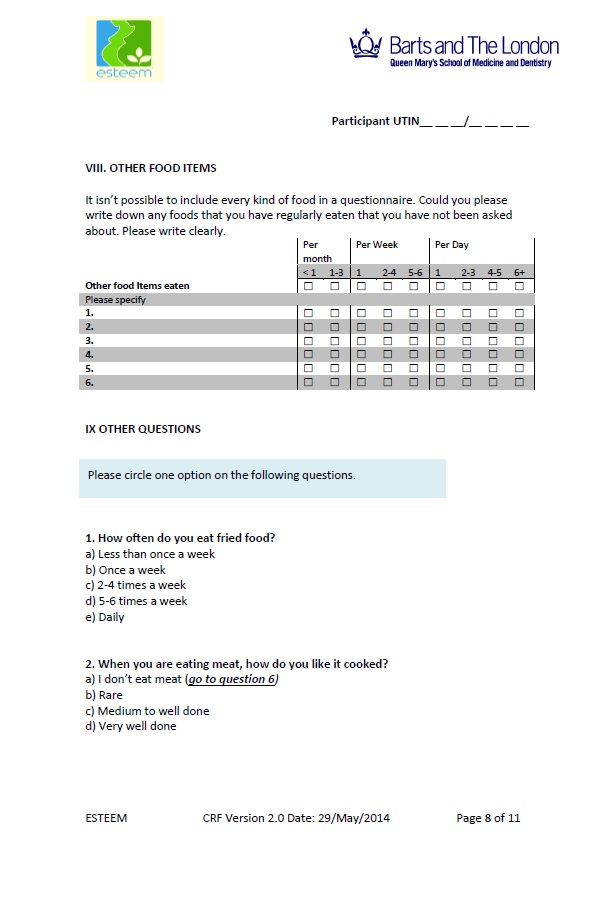
**

**
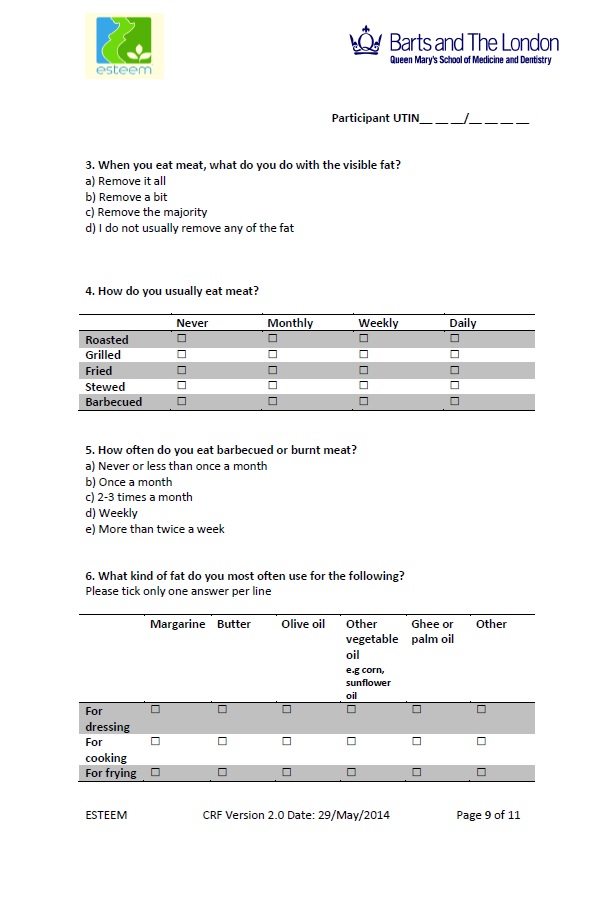

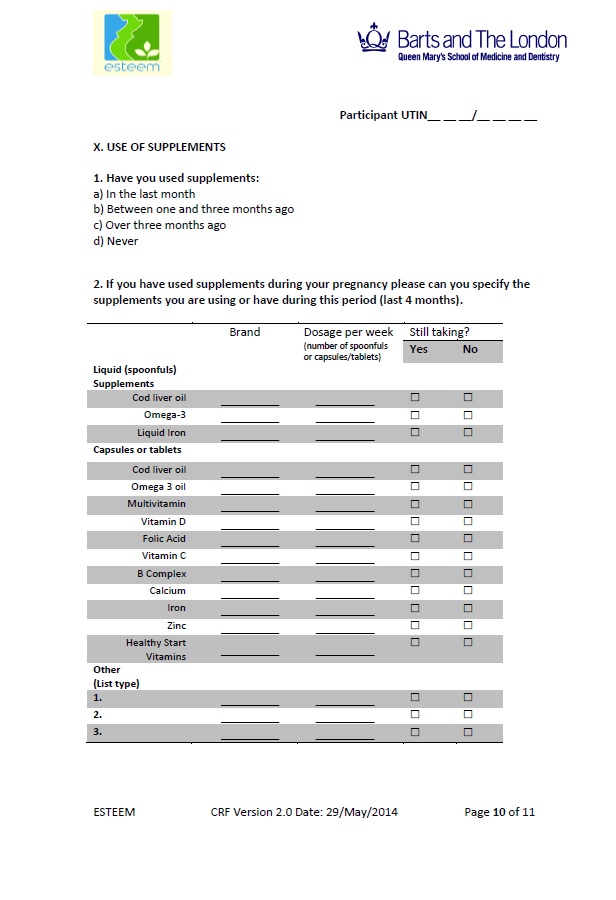
**

**
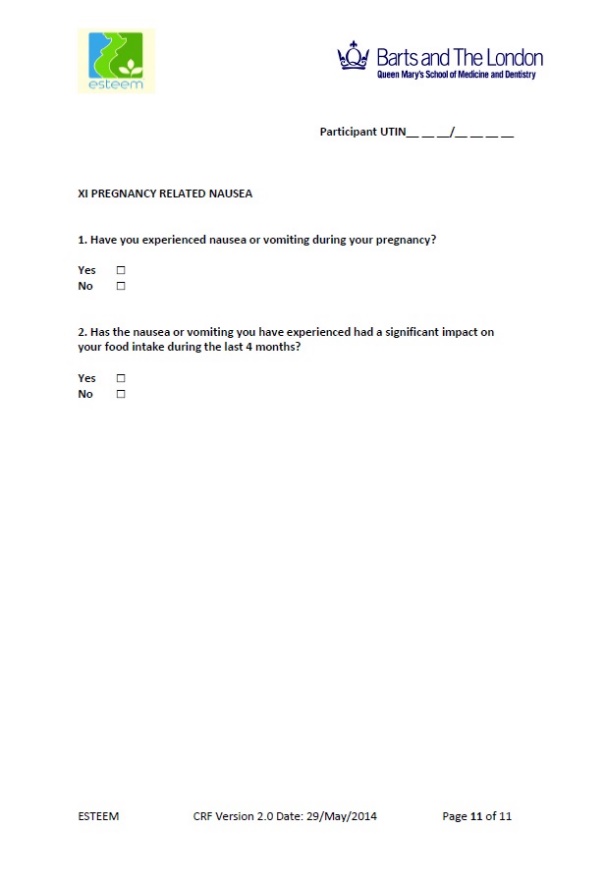
**

5E: 24-hour recall of dietary intake


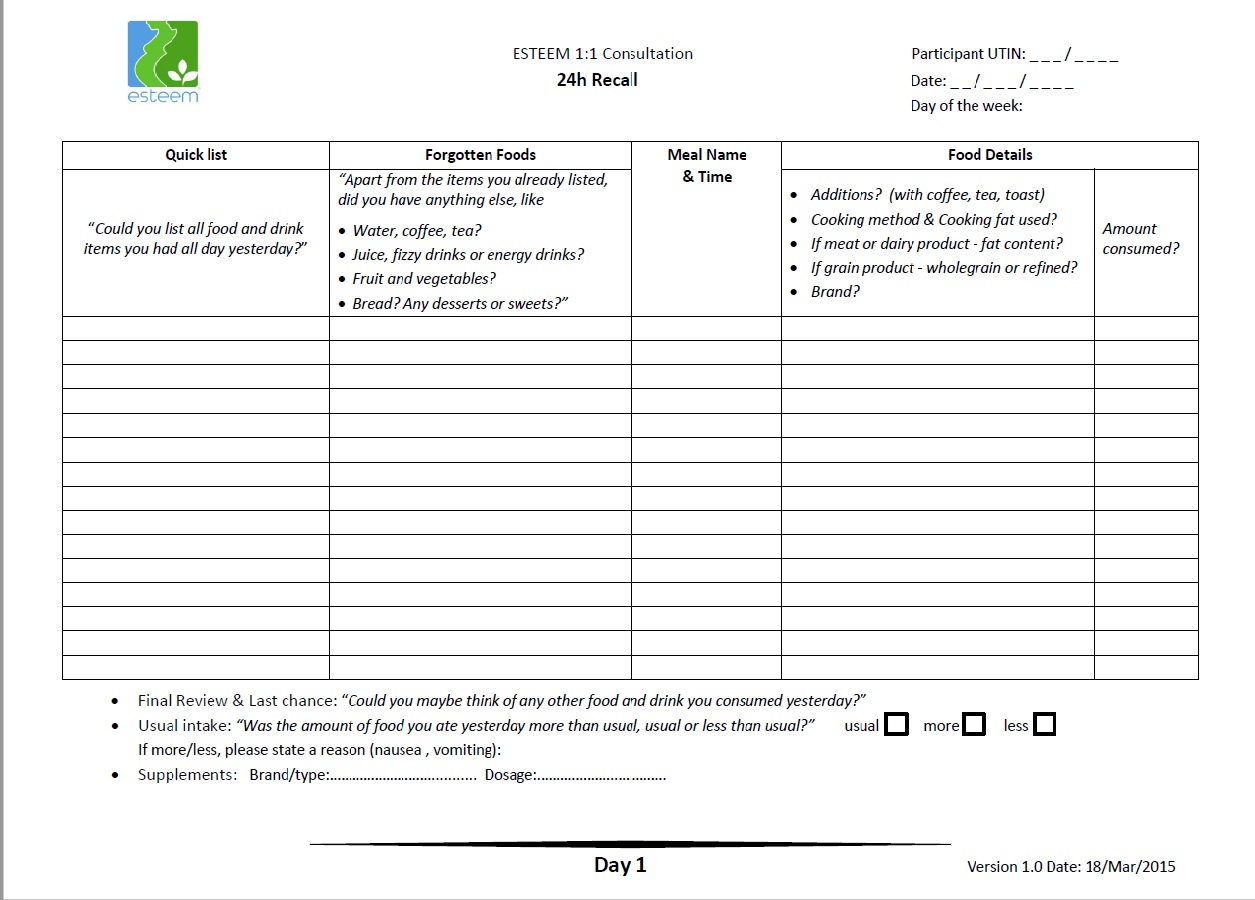

Supplement: S5 Text — ESTEEM Q, Effect of Simple, Targeted Diet in Pregnant Women With Metabolic Risk Factors on Pregnancy Outcomes questionnaire; FFQ, Food Frequency Questionnaire; IPAQ, International Physical Activity Questionnaires. (DOCX) [file pmed.1002857.s008.docx]
