# Supplementary material for: Mediterranean-style diet in pregnant women with metabolic risk factors (ESTEEM): A pragmatic multicentre randomised trial
Source: PLoS Med. 2019 Jul 23;16(7):e1002857. doi: 10.1371/journal.pmed.1002857 (PMC6650045; doi:10.1371/journal.pmed.1002857)
Supplement: S6 Text — (DOCX) [file pmed.1002857.s009.docx]

**S6 Text:** Definition of the primary outcomes

***6A. Composite maternal outcome combining gestational diabetes or pre-eclampsia***

**Gestational diabetes**

Defined as per the modified International Association of Diabetes and Pregnancy Study Groups (IADPSG) criteria—ie, fasting venous glucose of 5·1 mmol/L or higher or 2 h venous glucose of 8·5 mmol/L or higher, or a combination of these.

**Pre-eclampsia**

Either new onset or superimposed pre-eclampsia

*New Onset pre-eclampsia:*

New onset hypertension after 20 weeks gestation defined as systolic BP ≥ 140 mm Hg or diastolic BP ≥ 90 mmHg, in at least two readings, taken 4-6 hours apart plus new onset proteinuria defined as spot urine PCR test greater than 30mg/mmol or >24 hour urine 300mg/24 hours or 2+ or more on standard urinary dipstick tests after 20 weeks gestation.

*Superimposed pre-eclampsia in women with chronic hypertension or chronic proteinuria*:

In women with chronic hypertension and no proteinuria at baseline, the appearance of new onset proteinuria, (defined above) constitute a ‘superimposed pre-eclampsia.’ Chronic hypertension is hypertension that is present at the booking visit or before 20 weeks or if the woman is already taking antihypertensive medication at booking. In women who had proteinuria at base line, the diagnosis of superimposed preeclampsia requires an elevated serum alanine aminotransferase concentration (>70 U per litre) or worsening hypertension (either two diastolic BP of at least 110 mm Hg four hours apart or one diastolic measurement of at least 110 mm Hg or if the woman had been treated with an antihypertensive drug).

Women with eclamptic seizures with no hypertension or proteinuria are considered to have pre-eclampsia.

***6B. Composite offspring outcome combining stillbirth, small for gestational age (SGA) fetus, or admission to the neonatal intensive care unit.***

**Small for gestational age fetus**

Defined as birth weight less than 10th centile using customised growth charts taking into account gestational age at delivery, maternal height, weight, parity and ethnicity.

**Stillbirth**

Fetal death antepartum or intrapartum after 24 weeks’ gestation

**Admission to the neonatal intensive care unit**

Any condition that needs admission to the neonatal unit for further monitoring and management of the newborn.
